# Neuronal Cell Bodies Remotely Regulate Axonal Growth Response to Localized Netrin-1 Treatment via Second Messenger and DCC Dynamics

**DOI:** 10.3389/fncel.2016.00298

**Published:** 2017-01-05

**Authors:** Agata Blasiak, Devrim Kilinc, Gil U. Lee

**Affiliations:** ^1^Bionanosciences Group, School of Chemistry, University College DublinDublin, Ireland; ^2^UCD Conway Institute of Biomedical and Biomolecular Research, University College DublinDublin, Ireland

**Keywords:** microfluidics, pathfinding, guidance cues, compartmentalization, calcium signaling

## Abstract

Netrin-1 modulates axonal growth direction and speed. Its best characterized receptor, Deleted in Colorectal Cancer (DCC), is localized to growth cones, but also observed in the cell bodies. We hypothesized that cell bodies sense Netrin-1 and contribute to axon growth rate modulation, mediated by the second messenger system. We cultured mouse cortical neurons in microfluidic devices to isolate distal axon and cell body microenvironments. Compared to isolated axonal treatment, global Netrin-1 treatment decreased the axon elongation rate and affected the dynamics of total and membranous DCC, calcium, and cyclic nucleotides. Signals induced by locally applied Netrin-1 propagated in both anterograde and retrograde directions, demonstrated by the long-range increase in DCC and by the increased frequency of calcium transients in cell bodies, evoked by axonal Netrin-1. Blocking the calcium efflux from endoplasmic reticulum suppressed the membranous DCC response. Our findings support the notion that neurons sense Netrin-1 along their entire lengths in making axonal growth decisions.

## Introduction

The development of a functional nervous system requires the precise control of axonal growth at the single cell level. Axons are guided by biochemical and physical cues encountered by their leading tip, the growth cone, which converts external cues into axon turning and outgrowth decisions. Netrin-1, a member of the netrin family, is a key guidance molecule in the development of the nervous system as observed in knockout studies: mice lacking genes encoding Netrin-1 or its best characterized receptor, Deleted in Colorectal Cancer (DCC), suffer from acute neurodevelopmental failures (Serafini et al., 1996; Fazeli et al., 1997). Depending on the neuronal type, age, and location in the nervous system, Netrin-1 induces attraction, repulsion, accelerated outgrowth, or slowdown in developing axons (Bradford et al., 2009). Sensing of Netrin-1 by the growth cone activates the second messenger system, Ca^2+^ and cyclic nucleotides—cyclic adenosine monophosphate (cAMP) and cyclic guanine monophosphate (cGMP). Netrin-1 concentration gradient asymmetrically increases intracellular Ca^2+^ level ([Ca^2+^]_i_) across the growth cone and the axon changes its direction mediated by the initial level, as well as the source and extent of Ca^2+^ influx (Tojima et al., 2011). Ca^2+^ influx from the extracellular space alone results in repulsive turning, while simultaneous Ca^2+^ influx from the extracellular space and Ca^2+^ release from internal stores—primarily from the endoplasmic reticulum (ER) *via* ryanodine receptors (RyRs)—results in attractive turning (Hong et al., 2000). Axon turning is further modulated by cyclic nucleotides: decrease and increase of the cAMP:cGMP ratio result in repulsion and attraction, respectively (Nishiyama et al., 2003). Second messengers relocate DCC from the cytosol to the plasma membrane (Bouchard et al., 2004; Bartoe et al., 2006), which is likely to be the cause of the observed elevation of membranous DCC upon Netrin-1 treatment (Taylor et al., 2015). In contrast to extensive studies on axon turning, fewer efforts have focused on Netrin-1's effects on axon elongation rate. We recently showed that when applied locally to the axons, uniform concentrations of Netrin-1 increase membranous DCC level and slow down cortical axons (Blasiak et al., 2015). The roles of second messengers specific to these processes are poorly understood.

Immunostaining of Netrin-1 receptors revealed that they are not restricted to growth cones (Bouchard et al., 2008); however, their function in other subcellular regions is not clear. One possibility is that the receptors in the axon shaft and cell bodies mediate Netrin-1's effects that are unrelated to axon pathfinding. Netrin-1 plays various different roles in the nervous system, such as, precursor cell migration, synaptogenesis and cell-cell interactions (reviewed in Lai Wing Sun et al., 2011). In addition, its receptors, DCC and uncoordinated 5 (UNC5), act as dependence receptors, i.e., mediate or inhibit apoptosis in the absence or presence of a ligand, respectively (Mehlen and Furne, 2005; Furne et al., 2008). Alternatively, the cell bodies and axon shafts may be sensing Netrin-1 to cooperate with the growth cones in regulating the axon growth response. This would be consistent with the hypothesis that—complimentary to immediate and biased turning of growth cones—neurons are able to compare ligand concentrations along their lengths, i.e., between the growth cone and the cell body, to modulate their growth rates (Mortimer et al., 2010). Despite these observations, signaling mechanisms downstream of Netrin-1 acting on cell bodies has not been investigated.

The involvement of cell bodies in the control of axon growth rates would require communication with the growth cones. The mechanisms of long-range signal propagation in neurons include—among others—the modulation of second messengers, Ca^2+^ and cyclic nucleotides (Albus et al., 2013). The mechanism of action for the latter is not clear. Several observations favor the notion that Ca^2+^ waves propagate the cAMP signal, e.g., calcium can activate cAMP signaling in neurons as shown in the presence (Nicol et al., 2011) and in the absence of Netrin-1 (Gorbunova and Spitzer, 2002). Accordingly, Ca^2+^ waves propagate the retrograde signal downstream of Slit, another guidance molecule (Guan et al., 2007). Although axon-soma signal propagation mechanisms have been extensively studied, little is known about these processes in the context of Netrin-1 signaling.

In this study, we used a bicompartmental microfluidic neuron culture device to independently control subcellular microenvironments and dissected the role of somatic stimulation in the axonal growth response to Netrin-1. Our results show that local (isolated) and global Netrin-1 treatments differentially affect axonal elongation and the total and membranous DCC levels in different subcellular regions. Furthermore, Netrin-1 induces long-range effects, i.e., local Netrin-1 signal is anterogradely and retrogradely propagated, regulated through second messengers, Ca^2+^, cAMP, and cGMP.

## Materials and methods

### Cell culture and transfections

The animal handling procedures were approved by the UCD Animal Research Ethics Committee. Cortices were obtained from embryonic day 14 CD-1 Outbred mouse (*Mus musculus*; Charles River, Margate, UK) and neurons were dissociated as previously described. The dissection was performed in 10 mM Dulbecco's Phosphate Buffer Solution (DPBS; Lonza, Basel, Switzerland) with 0.1% glucose (Sigma, Wicklow, Ireland). Tissue collected from an entire litter (6–15 embryos) was mixed together, cut into smaller pieces, dissociated with Trypsin-EDTA (Gibco, Dun Laoghaire, Ireland) for 7 min at 37°C, and resuspended in DPBS with glucose. Trypsinization was blocked by adding 10% Fetal Bovine Serum (FBS; Gibco). The suspension was treated with DNAse (Invitrogen, Dun Laoghaire, Ireland) for 5 min, washed 3 × with DPBS and resuspended in DMEM-Glutamax (Gibco) supplemented with 1% Penicillin/Streptomycin (P/S; Gibco). The cells were mechanically dissociated, collected through centrifugation at 90.5 × g for 6 min, and resuspended in culture medium consisting of DMEM-Glutamax supplemented with 10% FBS and 1% P/S. For Förster Resonance Energy Transfer (FRET) imaging, cells were transfected with cGi500 (cGMP reporter) (Russwurm et al., 2007) or epac2-camp (cAMP reporter) (Nikolaev et al., 2004) DNA plasmids *via* electroporation (Neon Transfection System, Invitrogen). Briefly, 10^6^ cells and 15 μg plasmid was suspended in 100 μl transfection buffer and the cells were electroporated with an optimized protocol (three 10 ms pulses of 1500 V). The cell suspension was then transferred to warm (37°C) Neurobasal medium and centrifuged to bring its volume to *ca*. 35 μl, resulting in a density of 25–30 × 10^6^ cells ml^−1^.

Polydimethylsiloxane (PDMS) microfluidic devices were fabricated through two-step photolithography, as described previously (Kilinc et al., 2011, 2014). Devices were UV-sterilized for 30 min, incubated with 0.1 mg ml^−1^ Poly-l-Lysine (PLL; Sigma) in DPBS overnight at 37°C and rinsed with DPBS prior to cell seeding. Cells were plated in the somatic compartment by emptying all wells and adding 2 μl of cell suspension to the top well of the somatic compartment and 2 μl of medium to the top well of the axonal compartment. Final seeding density was approximately 8 × 10^5^ cells cm^−2^. Cells were let attach for 5 min and all wells were filled with culture medium containing B_27_ neuron supplement (Gibco). Devices were placed in Petri dishes containing 0.1% ethylenediaminetetraacetic acid (EDTA; Sigma) in water to minimize evaporation during incubation (37°C, 5% CO_2_). At 3 days *in vitro* (DIV), the culture medium was replaced with Neurobasal medium (Gibco) supplemented with 1% P/S and B_27_.

### Measurement of axon elongation

Live cell imaging was performed on 5–6 DIV. Neurons were locally or globally subjected to Netrin-1 (R&D Systems, Abingdon, UK). Media in both compartments were replaced with fresh or treatment-supplemented media, buffered with 25 mM HEPES (Sigma). Isolated treatment of a selected subcellular region was achieved by adding 5 μl less medium to the target compartment, i.e., axonal or somatic. 5–6 positions were chosen per coverslip to image as many single, non-overlapping growth cones as possible. The Petri dish containing the devices was placed on the motorized stage of an inverted microscope (Zeiss, Cambridge, UK) equipped with an EMCCD camera (Hamamatsu, Hertfordshire, UK) and an environmental chamber (Life Imaging Services, Basel, Switzerland), which maintained the temperature at 37°C. The microscope was controlled with Axiovision software (Zeiss, Cambridge, UK). Brightfield images were taken with a 40 × 0.6 NA objective every 10 min from 25 ± 5 min to 95 ± 5 min following the treatment. The early and late measurements include the data collected from 25 ± 5 min to 55 ± 5 min and 65 ± 5 min to 95 ± 5 min periods after the treatment, respectively. Axon elongation was analyzed in terms of speed, i.e., displacement between consecutive time-lapse images divided by time, and velocity, i.e., the position vector between the initial and final positions of the growth cone divided by time. Only one experimental condition was tested in each culture. The data for each condition were collected from at least three independent cultures with cells obtained from at least two litters. All single, non-overlapping growth cones in the first time frame were chosen for the analysis. MTrackJ plug-in of ImageJ software (National Institutes of Health, Bethesda, MD) was used to track the movement of growth cones (Meijering et al., 2012).

### Immunocytochemistry and quantification of DCC

Cells on 5–7 DIV were incubated with Netrin-1 for 1, 5, 25, or 90 min after replacing the media in all wells at the same time. To block RyRs, axonal medium was supplemented with 100 μM ryanodine (Calbiochem, Carrigtwohill, Ireland; High Ry) prior to and during axonal Netrin-1 treatment. Following the incubation cells were fixed with 0.5% Gluteraldehyde (Sigma) in PBS (Sigma) for 15 min at room temperature (RT) and subsequently rinsed with PBS. Immunocytochemistry in the microfluidic devices followed similar protocols as for the cultures on glass slides (Glynn and McAllister, 2006). The solutions were introduced to only one well of each compartment to guarantee pressure-driven flow through the compartments. Cells were treated for 30 min with a blocking buffer composed of 1% Bovine Serum Albumin (BSA; Sigma) in PBS. To determine the total levels of DCC, cells were treated with the blocking buffer containing 0.5% Triton X-100 (Sigma). Blocking buffer without Triton X-100 was used to immunostain membranous DCC. Cells were incubated with primary antibodies for 1 h in RT: 1:100 mouse anti-DCC against the extracellular domain (Clone AF5; Calbiochem, OP45, Lot: D00129946) or 1:300 mouse anti-β-III-tubulin (Promega, Kilkenny, Ireland). After washing with PBS cells were incubated with Alexa568 Phalloidin (1:200; Invitrogen) and Alexa488 goat anti-mouse (1:300; Invitrogen, A11029, Lot: 716811) for 1 h in RT. Finally, cells were counterstained with Hoechst 33342 (Invitrogen) for 15 min at RT, and the wells were topped with Mowiol (Sigma). To quantify the change in receptor levels upon Netrin-1 treatment, control and experimental cultures were prepared under identical conditions in separate circuits bonded on the same coverslip. Imaging was performed using an inverted, epi-fluorescent microscope (Olympus, Southend-on-Sea, UK) equipped with a CCD camera (Hamamatsu). All micrographs used for quantification were taken using the same time after staining, microscope, objective, and exposure time to allow for direct comparison of the measurements. Somatic and axonal compartments were imaged in DAPI, FITC, and TRITC channels. Regions of interest (ROIs) were selected based on Phalloidin (axons) or Hoechst (cell bodies) staining: blindly to the signal intensity in the green channel. Approximately 100 ROIs were selected from at least 3 independent devices for each condition. Each ROI represents a single, individual cell. Cell body clusters were not included in the analysis. Growth cone ROIs represent the centers of growth cones. Filopodia were defined as linear structures emanating from growth cones that have high actin staining intensity. Their length was between 1.90 and 6.06 μm. A single filopodium ROI was analyzed per growth cone, which sufficiently represented DCC:Phalloidin staining intensity ratio, as confirmed by Wilcoxon Rank Sum test comparing data obtained from a single filopodium per growth cone and from multiple filopodia per growth cone (*n* = 15 growth cones; *p* > 0.70 for controls; *p* > 0.56 for axonal Netrin-1 treated). The cell bodies' ROIs did not discriminate between neurons with or without an axon crossing to the axonal compartment. Mean pixel intensity for each ROI was measured in each channel using ImageJ software. Background signal intensity, defined as the intensity of the cell-free area neighboring the ROI, was subtracted from the signal intensity of the ROI in both channels. The signal intensity of total DCC was normalized with Phalloidin signal intensity obtained from the same ROI and with the signal intensity of the control group from the same imaging session; the signal intensity of the membranous receptor was normalized with the membranous receptor signal intensity of the control group from the same imaging session. All images within each figure are presented with identical intensity adjustments. In a subset of experiments, Calcein AM (Cayman Chemical, Ann Arbor, MI) was used to identify cell bodies sending axons to the axonal compartment. Axons in the axonal compartment were incubated with 1 mM Calcein AM for 30 min. Green fluorescence was imaged prior to fixation to identify Calcein-labeled cells. Fixed cells were stained with Hoechst for 15 min. Images taken prior and after cell fixation were manually matched in ImageJ. The datasets with membranous DCC signal intensities in the cell bodies after axonal Netrin-1 treatment were presented as data histograms, whose bin-widths were determined using the method by Shimazaki and Shinomoto (2007), applied to the control dataset.

### FRET imaging and analysis

Dynamic changes in cGMP and cAMP were measured using transfected neurons cultured in microfluidic devices for 7 days. The media in all wells were replaced with 20 μl imaging medium (Neurobasal without phenol red, with 1% P/S, B_27_ and 25 mM HEPES). 10 μl high viscosity dimethylpolysiloxane (Sigma) was added to each well to reduce evaporation during imaging. A single PDMS pad with up to three microfluidic devices was imaged using the same microscope and camera described for axon elongation experiments using a 100 × 1.46 NA objective and a xenon arc lamp (Lambda LS/30, Sutter Instruments, Novato, CA). Two filter sets (Semrock, Rochester, NY) consisting of the same excitation filter (438/24 nm) and dichroic mirror (458 nm), but alternative emission filters, 483/32 nm or 542/27 nm for CFP and YFP fluorescence, respectively, were used in tandem. Snapshots in both channels were taken every 10 s for 10–15 min, where the time period between cyan and yellow channels due to filter wheel rotation is estimated to be 0.5 s. 10 μl of 5 μg ml^−1^ Netrin-1 in imaging medium was added to one of the wells after *ca*. 10 time points after the start of imaging, without touching the coverslip. The final Netrin-1 concentration in the treatment compartment was about 1 μg ml^−1^. To test control conditions, 10 μl of fresh imaging medium without Netrin-1 was introduced to one of the wells. Signal analysis was performed using ImageJ. Treatment application caused a short-term loss of focus—these initial time-points were excluded from the analysis. Occasionally, time points affected by a focus shifts in the later stages of imaging (85 instances in >6500 data points) were excluded from the analysis. Background signal intensity was subtracted from the signal in each channel for measurements in growth cones. The 15 min-long time-lapse imaging showed that signal intensities in each channel exhibited a logarithmic decrease due to photo-bleaching and channel bleed-through; therefore, we corrected the signal in each channel with a logarithmic function whose coefficients were calculated based on the intensity profile (for that channel) prior to the treatment. The intensity profile also depended on the initial signal intensity, which varied from neuron to neuron, as a function of transfection quality. Data were presented as the CFP:YFP background (BG)-subtracted fluorescence intensity ratio: ΔR = (CFP − BG_CFP_)/(YFP − BG_YFP_) (Börner et al., 2011), normalized by the same ratio before Netrin-1 application (R). Standard error of the mean (s.e.m.) was calculated for each time point from data acquired from 5 to 9 measurements.

### Calcium measurements

Neurons at 5–6 DIV were incubated with 4 μM of Fluo4 AM calcium dye (Invitrogen) for 20–40 min. After dye loading, cells were washed with 7 μl medium added to one well of each compartment, followed by a 30–40 min incubation at 37°C. Prior to imaging, media in each well were replaced with 20 μl imaging medium and topped with 10 μl dimethylpolysiloxane. The experimental procedure was the same as in FRET measurements. For the measurements in the cell bodies, only the neurons up to 50 μm away from the microchannels were chosen for the analysis to ensure they had axons in the axonal compartment; 75–90% of the cell bodies fulfill this assumption as shown by the Calcein AM stains (**Figures 3A,B**). Fluorescent time-lapse images were acquired every 10 s for 25 min. Images were acquired with reduced excitation light intensity to minimize phototoxicity. Calcium concentration was quantified as the background-subtracted average pixel intensity (F) in a ROI divided by the background-subtracted baseline level (F0) in the same ROI. Baseline was determined by fitting a straight line to the intensity data. To minimize the effect of Ca^2+^ transients on the fitting, signal intensities exceeding 115% of the fit line were excluded and the fitting was repeated. Calcium transients were defined as fluorescence intensity ratios (F/F0) exceeding 120% of the baseline level, confirmed by frame-by-frame analysis of the time-lapse images. We observed that *ca*. 6% of the cell bodies imaged had a very high frequency of Ca^2+^ transients prior to the treatment compared to the rest (20–70 h^−1^ vs. 1–3 h^−1^). These were classified as outliers and were excluded from the analysis.

### Statistical analysis

At least three independent experiments were performed for each assay. Statistical tests were performed using Matlab (MathWorks, Natick, MA). Minimum sample size (*n*) for each test was determined using the power analysis function in Matlab for a power value of 0.9. Lilliefors test was used to determine if the datasets were normally distributed. Non-normal, independent data were compared by Kruskal-Wallis test and Wilcoxon rank sum test with Dunn-Sidak *post-hoc* correction. Non-normal, dependent samples were compared by Wilcoxon sign-rank test. The distributions of membranous DCC signal intensities in the cell bodies obtained at different time points after axonal Netrin-1 treatment were compared with each other using the Kolmogorov-Smirnov test. Dunn-Sidak correction was applied if the number of comparisons exceeded two. Data are given as mean ± s.e.m.

## Results

### The treatment of cell bodies alters the elongation response of distal axons to Netrin-1

**Figure 1:**
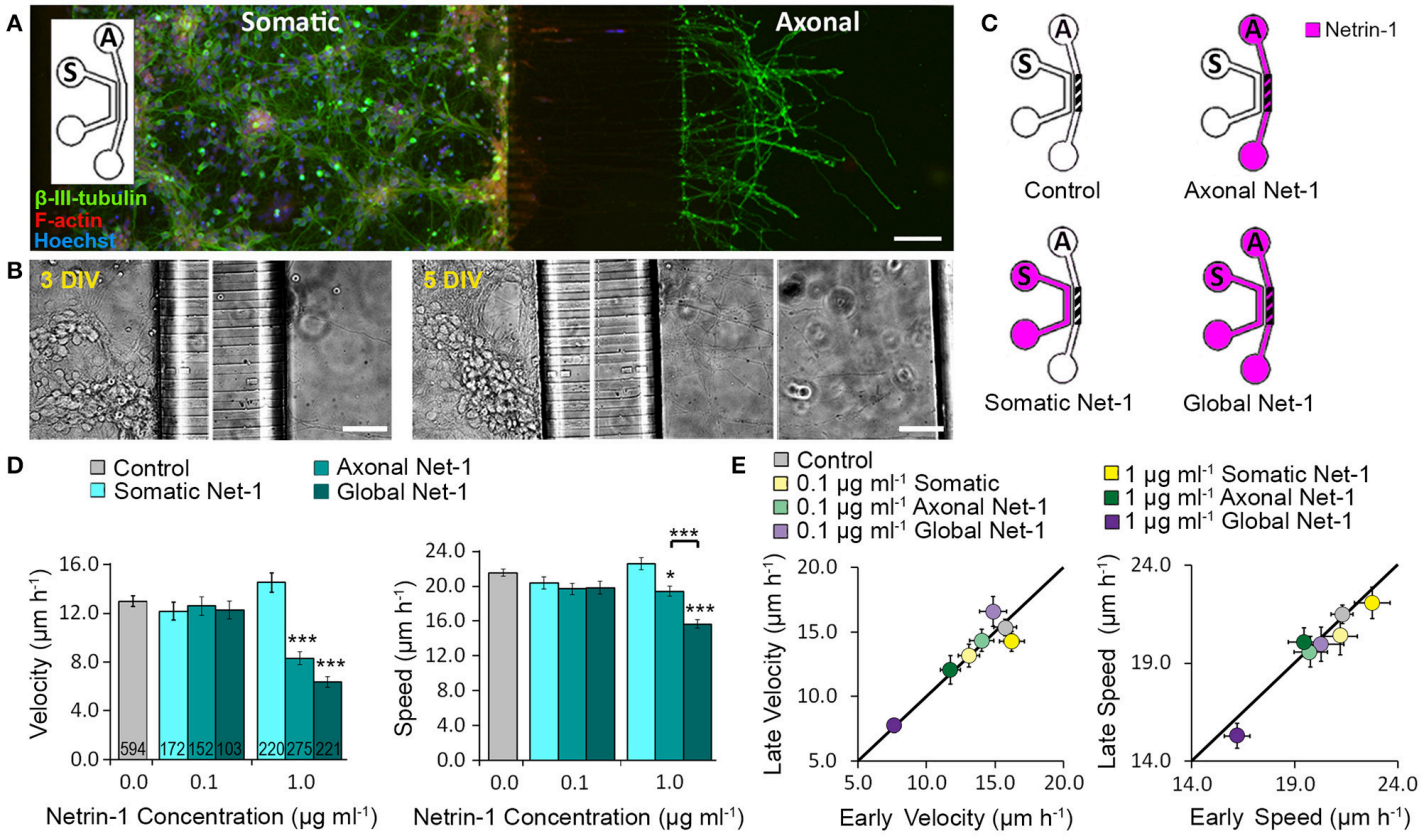
**Axon elongation depends on the localization of Netrin-1 treatment. (A)** Neurons cultured in the bicompartmental device (inset) send neurites from the somatic (*S*) to the axonal (*A*) compartment through microchannels. Scale bar = 80 μm. **(B)** Neuronal growth profile at 3 and 5 days *in vitro* (DIV). Scale bars = 50 μm. **(C)** Modes of Netrin-1 treatment. The axon elongation was measured in the axonal compartment (hatch). **(D)** Average velocity and speed (mean ± s.e.m.) for increasing Netrin-1 concentrations and different compartments of delivery. The numbers on bars represent the number of individual axons from *N* ≥ 2 independent experiments (Table S1); statistical significance compared to controls unless indicated otherwise; ^*^p < 0.05, ^***^p < 0.001. **(E)** Comparison of axon growth in the first (early) and last (late) 0.5 h of imaging. Data for axonal Netrin-1 treatment are re-presented with permission from Blasiak et al. (2015). Copyright 2015 American Chemical Society.

We cultured mouse cortical neurons in bicompartmental microfluidic devices (Figure 1A) that permit isolated, subcellular treatments. These devices consist of axonal and somatic compartments that are fluidically isolated for at least 4 h, as demonstrated using a fluorescent tracer (Blasiak et al., 2015). Axons reached the axonal compartment after 3 days and extended toward its distal wall after 5–6 days (Figure 1B). At this time, medium with 0.1 or 1.0 μg ml^−1^ Netrin-1 was added in either the somatic or the axonal compartment (somatic and axonal treatments, respectively), or simultaneously to both compartments (global treatment) (Figure 1C). Netrin-1 did not evoke growth cone collapse or axon retraction. Neuronal response was evaluated by characterizing distal axon elongation in the axonal compartment during 90 min of treatment in terms of velocity—the vector between initial and final positions of the growth cone divided by time, and speed—average instantaneous velocity measured every 10 min (Figure 1D). At low concentration (0.1 μg ml^−1^), Netrin-1 failed to affect axon elongation. At high concentration (1.0 μg ml^−1^; Movie S1), somatic Netrin-1 treatment did not affect the axon elongation; whereas, the axonal treatment significantly decreased axon velocity and slightly affected axon speed (64 and 90% of the control, respectively), meaning that the growth cone covered similar distance but progressed less. Global treatment severely decreased both, velocity and speed (49 and 72% of the control, respectively). These effects were consistent over time as shown by comparing axon elongation in the early (25 ± 5 min to 55 ± 5 min) and late (65 ± 5 min to 95 ± 5 min) periods of the treatment (Figure 1E). This analysis (based on 30 min intervals) did not reveal any velocity changes after axonal treatment, but clearly showed the more profound effect of the global treatment. The differential effects of Netrin-1 after axonal, somatic, and global treatments indicate that cell bodies sense Netrin-1 and contribute to the distal axonal response.

### Netrin-1 induces local and global changes in membranous DCC dynamics

**Figure 2:**
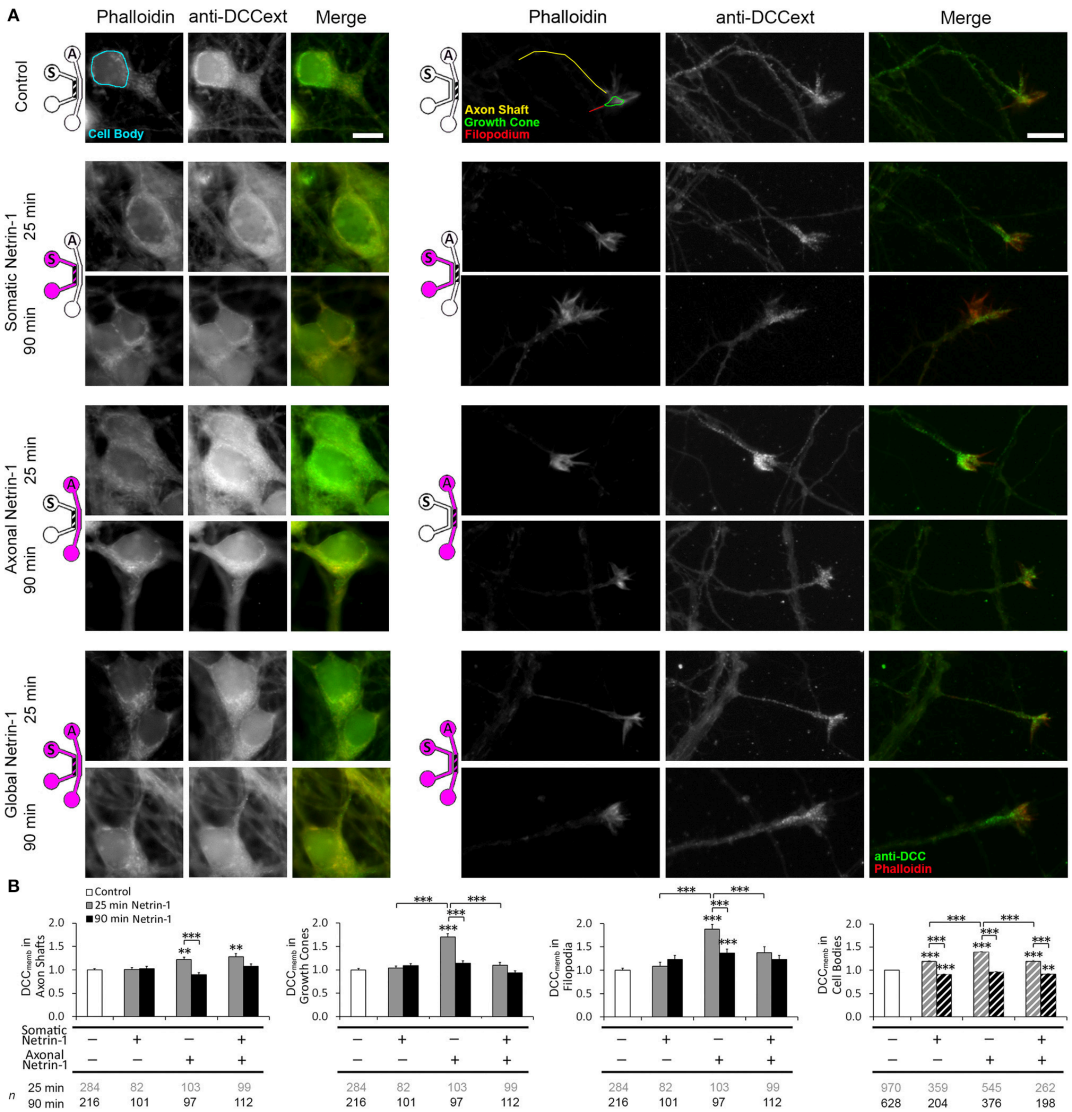
**Netrin-1 modulates local and long-range DCC insertion into plasma membrane. (A)** Membranous DCC staining in the control group and 25 and 90 min after isolated (axonal and somatic) or global Netrin-1 treatment. Schematics show which compartment, somatic (*S*) or axonal (*A*), underwent Netrin-1 treatment (pink) and which compartment was imaged (hatch). The ROIs were chosen in Phalloidin channel as shown by the color overlay in control. Scale bars = 10 μm. **(B)** Staining intensity (mean ± s.e.m.) was measured in the axonal compartment (solid fill) and in the somatic compartment (hatch fill), and normalized with the control signal. *n* is the number of individual ROIs from *N* ≥ 3 independent experiments (Table S2); statistical significance compared to controls unless indicated otherwise; ^**^p < 0.01, ^***^p < 0.001. Axonal compartment data for axonal Netrin-1 treatment are re-presented with permission from Blasiak et al. (2015). Copyright 2015 American Chemical Society.

Netrin-1 modulates DCC cycling on and off the plasma membrane (Kim et al., 2005), which has a direct effect on axon elongation (Bouchard et al., 2008). We tested how exposing different subcellular regions to Netrin-1 affects DCC dynamics in distal axons. Neurons were subjected to axonal, somatic, or global 1.0 μg ml^−1^ Netrin-1 by replacing the media in all wells of the somatic and axonal compartments at the same time, such that the pressure-driven flow through each compartment was minimal and no flow was induced from one compartment to the other (Blasiak et al., 2015). After 25 or 90 min long incubation, the cells were fixed and immunostained without cell membrane permeabilization (Piper et al., 2005). Membranous DCC signal (DCC_memb_) was present in all subcellular regions (Figure 2). Somatic treatment failed to affect DCC_memb_ in distal axons. Global treatment increased DCC_memb_ in distal axon shafts at 25 min, which returned to control levels at 90 min, but did not affect DCC_memb_ in growth cones or in filopodia. These effects are in striking contrast with the axonal treatment, which induces an initial (25 min) increase in DCC_memb_ in axon shafts, growth cones, and filopodia, which, in filopodia, is sustained for as long as 90 min (Blasiak et al., 2015). Observed changes in DCC_memb_ were not accompanied by changes in growth cone size: mean growth cone area after axonal Netrin-1 was 6.58 ± 0.26 μm^2^ at 25 min and 6.66 ± 0.32 μm^2^ at 90 min, compared to 6.65 ± 0.31 μm^2^ in controls. The difference between axonal and global treatments in the membranous DCC response in distal axons suggests long-range effects of exposing cell bodies to Netrin-1.

We next investigated if DCC on cell body membranes exhibited similar dynamics upon Netrin-1 treatment as in distal axonal regions. The majority of neurons did not have an axon in the axonal compartment; thus, they experienced somatic treatment as a global treatment rather than local. Accordingly, somatic and global treatments had the same effect on DCC_memb_ in cell bodies: it was elevated at 25 min and decreased below control levels at 90 min. Interestingly, axonal treatment, where Netrin-1 was absent from the somatic compartment, caused a more pronounced increase in DCC_memb_ in cell bodies at 25 min and returned to control levels at 90 min (Figure 2B). This observation suggests that Netrin-1 signal induced in distal axons propagated to their cell bodies.

### Long-range retrograde Netrin-1 signal increases DCC_memb_ within minutes

**Figure 3:**
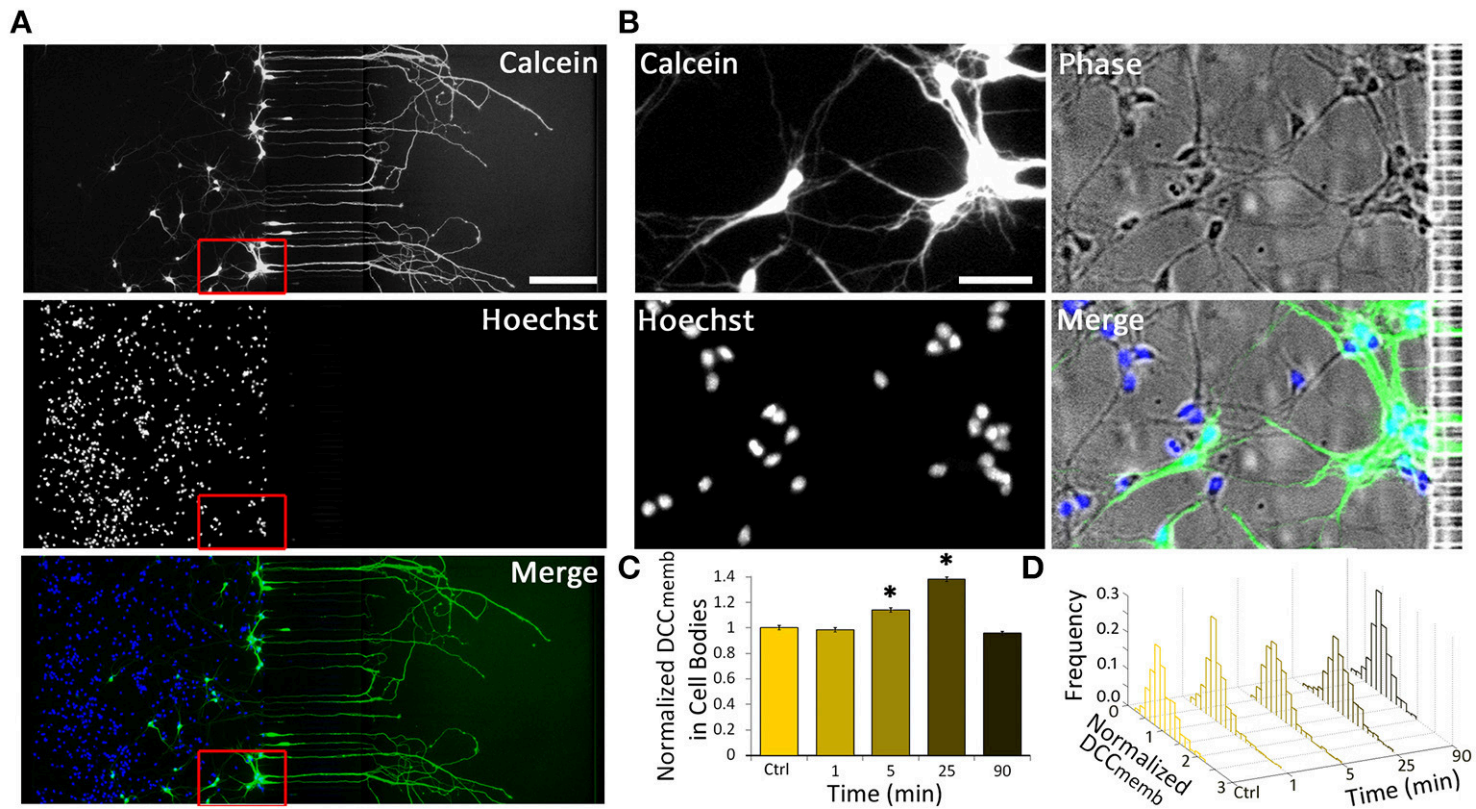
**Axonal Netrin-1-induced change in membranous DCC in cell bodies is uniformly distributed within the somatic compartment. (A)** Cell permeable Calcein AM labels entire neurons upon its uptake in the axonal compartment (green). Majority of neurons do not have an axon in the axonal compartment, as indicated by the nuclear stain (blue). Scale bar = 200 μm. **(B)** The boxed area in **(A)** is magnified to reveal neurons labeled and not labeled with Calcein near microchannels. Scale bar = 50 μm. **(C)** Membranous DCC staining intensity in cell bodies normalized by the control value for increasing duration of 1.0 μg ml^−1^ axonal Netrin-1 treatment. Error bars represent s.e.m.; *n* > 200 for each time point from *N* ≥ 3 distinct cultures (Table S3); statistical significance compared to controls with Kolmogorov-Smirnov test with Dunn-Sidak correction for multiple comparisons; ^*^p < 0.05. **(D)** Membranous DCC signal intensity distribution for each time point.

Intrigued by the bidirectional regulation of DCC membrane dynamics, we aimed to determine the speed and the extent of the effect of 1.0 μg ml^−1^ axonal Netrin-1 treatment on cell bodies (Figure 3). No change in DCC_memb_ was observed in cell bodies after 1 min-long treatment; however, at 5 min, DCC_memb_ was significantly higher. Although this value was lower than the peak signal at 25 min time point (Figure 3C), it shows that the effect of axonal Netrin-1 had reached cell bodies within 5 min. DCC_memb_ in cell bodies was quantified using images taken at randomly chosen areas in the somatic compartment. Thus, not all cell bodies analyzed had an axon in the axonal compartment, where the treatment was applied. To see how many neurons had neurites in the axonal compartment we labeled the axons in the axonal compartment with Calcein AM, a cell-permeant fluorescent dye. Within 30 min, Calcein stained entire lengths of neurons and was detected in 9–30% of cell bodies in the somatic compartment (Figures 3A,B), indicating the fraction of neurons with axons in the axonal compartment. Interestingly, when DCC_memb_ data (collected from cell bodies with and without an axon in the axonal compartment) were presented as histograms a uniform response was observed, i.e., histograms had the same shape for all time points during axonal Netrin-1 treatment (*p* > 0.05, Kolmogorov-Smirnov test with Dunn-Sidak correction) (Figure 3D). The mean DCC_memb_ increase followed by a decrease is evident from the shifting of the histograms to right and back to left over time. We further sought if DCC_memb_ in cell bodies depended on their distance from the microchannels, i.e., the probability of having an axon in the axonal compartment. No such correlation was detected when cell bodies were categorized into three regions with 250 μm increments from microchannels (ANOVA; *p* > 0.90 for 25 min time-point). Therefore, independent of their position in the somatic compartment, cell bodies exhibited increased DCC_memb_ after 25 min-long axonal Netrin-1 treatment (Figure 3 and Figure S1), consistent with the homogeneous distribution of DCC_memb_ signal among all cell bodies (Figure 3D).

### Netrin-1 induces local and global changes in total DCC levels

**Figure 4:**
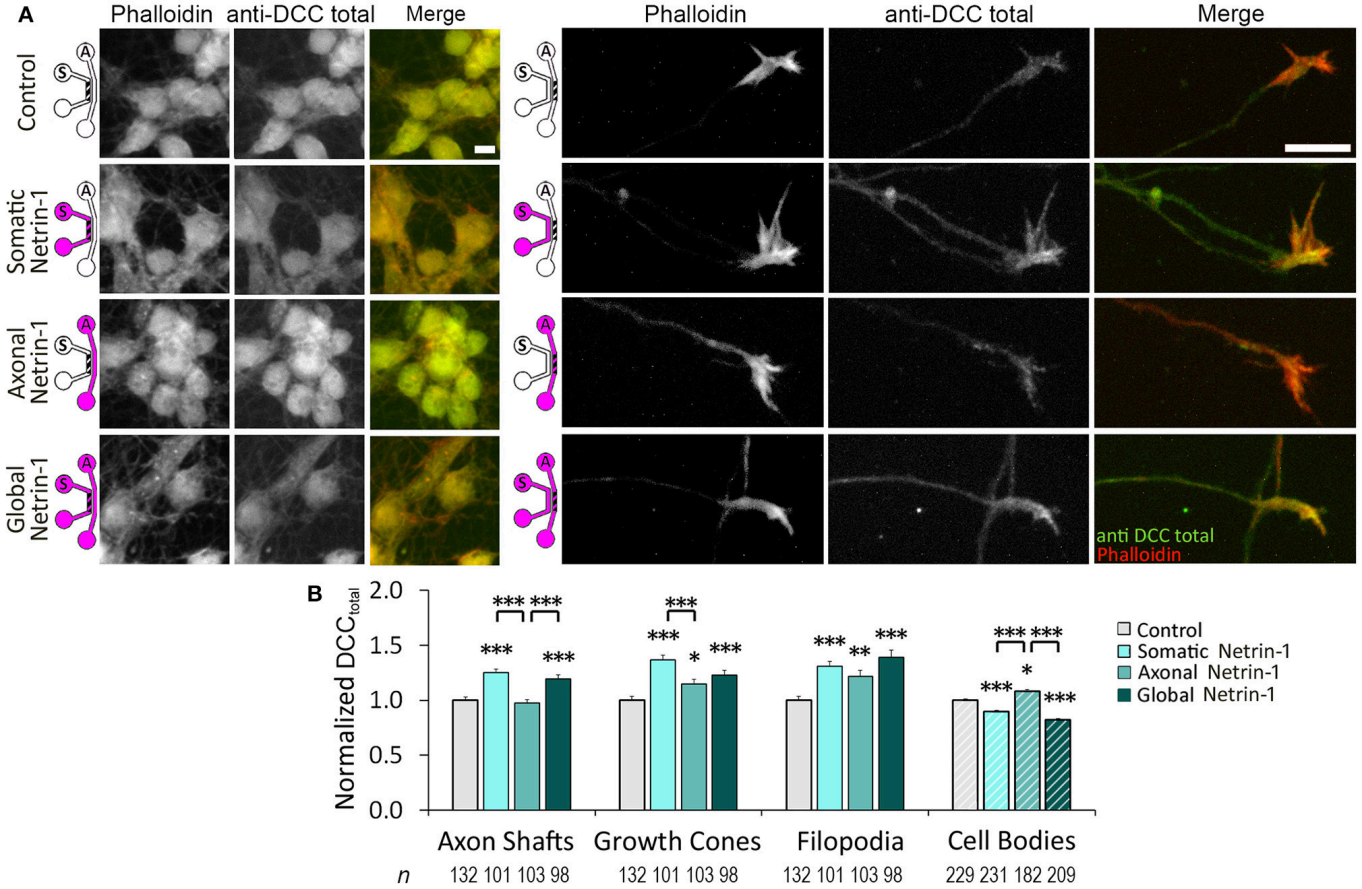
**Netrin-1 treatment modulates local and long-range total DCC. (A)** Total DCC staining upon 90 min-long somatic, axonal, or global treatment with 1.0 μg ml^−1^ Netrin-1 or vehicle (control). The schematics show which compartment, somatic (*S*) or axonal (*A*), underwent Netrin-1 treatment (pink) and which compartment was imaged (hatch). Scale bars = 10 μm. **(B)** DCC staining intensity (mean ± s.e.m.) was measured in the ROIs in the axonal (solid fill) and somatic (hatch fill) compartments, and normalized with the control signal. *n* is the number of individual ROIs from *N* ≥ 3 independent experiments (Table S4); statistical significance compared to controls unless indicated otherwise; ^*^p < 0.05, ^**^p < 0.01, ^***^p < 0.001. Axonal compartment data for axonal Netrin-1 treatment are re-presented with permission from Blasiak et al. (2015). Copyright 2015 American Chemical Society.

We next investigated if Netrin-1 also affected total DCC levels (DCC_total_) (Figure 4A). Neurons were fixed after 90 min of Netrin-1 (or vehicle) treatment and stained against DCC after membrane permeabilization. Total DCC staining was present in all subcellular regions, but was less punctate compared to membranous DCC staining. Somatic Netrin-1 treatment significantly increased DCC_total_ (normalized with the control value) in all distal axonal regions (Figure 4B). These increases were higher than those reported for DCC_total_ upon local axonal Netrin-1 treatment in distal axonal regions (Blasiak et al., 2015). Global Netrin-1 treatment had the same effect as the somatic treatment, i.e., the DCC_total_ increase in distal axonal regions after somatic and global Netrin-1 treatments were statistically not different. In the cell bodies, Netrin-1 induced a very different DCC_total_ response: somatic and global Netrin-1 treatments decreased DCC_total_, while axonal Netrin-1 treatment increased DCC_total_. There was no difference in the DCC_total_ signal across the somatic compartment (ANOVA; *p* > 0.44; Figure S2).

### Netrin-1 regulates dynamics of cyclic nucleotides

Crosstalk between second messengers, cyclic nucleotides and Ca^2+^, plays a crucial role in axon pathfinding (Nicol et al., 2011; Averaimo and Nicol, 2014) and modulates the axonal response to Netrin-1 (Hong et al., 2000). We investigated the effects of somatic, axonal, and global Netrin-1 treatments on cyclic nucleotides activity in growth cones and cell bodies with genetically encoded FRET reporters: cGMP reporter cGi500 (Russwurm et al., 2007) or cAMP reporter epac2-camp (Nikolaev et al., 2004). These reporters consist of two chromophores, energy donor and acceptor, where the energy transfer occurs when the chromophores are in close proximity. Therefore, the measurement of the donor and acceptor emission intensities after exciting the donor is representative of the distance between the two. In the inactive state of the reporters the chromophores are close to each other (hence high signal from the acceptor), but, upon binding to cyclic nucleotides, their conformation change and the distance between the chromophores increases, resulting in a detectable drop in the acceptor's and increase in the donor's intensities. The increase (or decrease) of the donor:acceptor signal intensity ratio is therefore representative of the increase (or decrease) in the cyclic nucleotide levels. Reporter specificity was confirmed *via* bath-treating neurons with 10 μM forskolin, a known cAMP enhancer (Nairn et al., 1985), which elevated (ΔR/R)_cAMP_ to 1.2 × in the Epac1-camp transfected neurons (Figure S3C). Transfected neurons with bright fluorescence with (Figures S3A,B) and without an axon crossing to the axonal compartment were selected for local and global Netrin-1 treatments, respectively. To facilitate the visualization of neuronal response within seconds after subcellular stimulation, the treatment was rapidly delivered to the target compartment by inducing a pressure-driven flow through this compartment. We observed that vehicle delivery to the somatic chamber affected the cell body signal, i.e., (ΔR/R)_cGMP_ increased to 1.1 × and (ΔR/R)_cAMP_ decreased to 0.8 × control value. This effect was absent when vehicle was delivered globally without inducing flow, i.e., to neurons cultured on a glass-bottom Petri dish without the microfluidic device (Figure S3D); we thus attribute the changes in cAMP and cGMP levels to fluid shear stress acting on cell bodies. Vehicle delivery to either compartment did not affect the growth cone signal.

**Figure 5:**
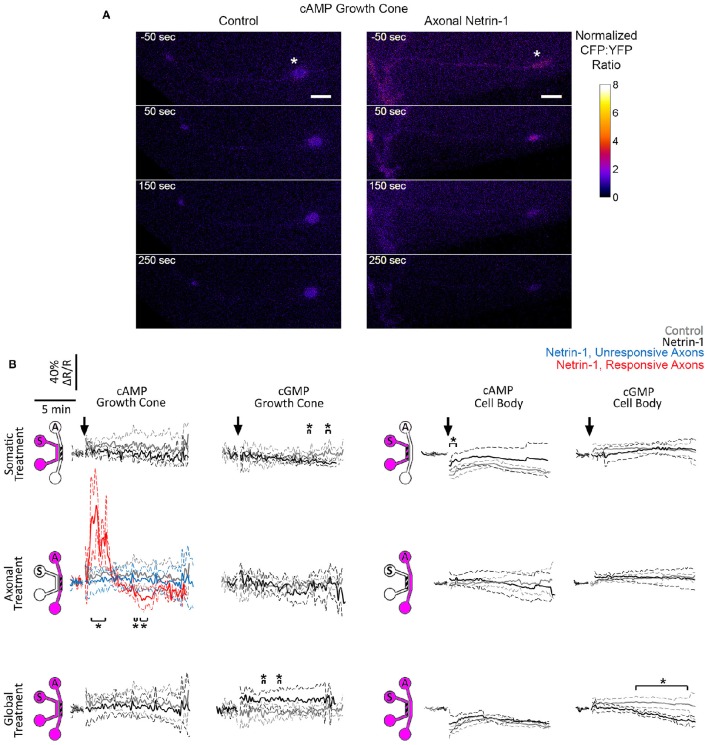
**Netrin-1 modulates cyclic nucleotides differentially for the local and global treatments. (A)** Pseudo-colored cAMP CFP:YFP fluorescence intensity ratio (ΔR), normalized by the baseline ratio (R), in distal axons in response to axonal treatment with Netrin-1 or vehicle (control). Scale bars = 5 μm. **(B)** cAMP and cGMP signals in growth cones and in cell bodies in response to treatments (arrows) with vehicle (gray) or with local or global Netrin-1 treatments (black). Growth cone cAMP signals are given separately for responsive (red) and unresponsive (blue) axons. Data are given as mean with 95% confidence interval (broken lines). ^*^p < 0.05. Measurements were done in *N* ≥ 2 distinct cultures (Table S5). For individual data traces, see Figure S4.

We first measured cyclic nucleotide levels in growth cones (Figure 5, Figure S4). Somatic Netrin-1 treatment did not affect the (ΔR/R)_cAMP_ or (ΔR/R)_cGMP_ levels. Axonal Netrin-1 treatment did not affect (ΔR/R)_cGMP_, but induced a rapid increase in (ΔR/R)_cAMP_, which reached 1.87 × control value at 2 min and returned to control levels by 4 min (Figure 5). 3 out of 7 tested axons remained non-responsive, which is in agreement with the presence of neuronal subpopulations with different responsiveness to Netrin-1 (Blasiak et al., 2015). In contrast, global Netrin-1 treatment slightly increased (ΔR/R)_cGMP_ (no statistical significance), but did not affect (ΔR/R)_cAMP_, suggesting that the co-stimulation of cell bodies suppressed the effect of local axonal stimulation and blocked the rise in cAMP in growth cones. We next measured cyclic nucleotide activity in cell bodies. Netrin-1 did not cause any apparent changes in cyclic nucleotide levels for any of the treatment modes: although statistical analysis indicated that somatic Netrin-1 induced an early, brief rise in (ΔR/R)_cAMP_ and that global Netrin-1 induced a late, slight decrease in (ΔR/R)_cGMP_ level (both relative to controls), the magnitudes of these changes were too small to draw conclusions. Cumulatively, our cyclic nucleotide measurements suggest that the localization of Netrin-1 treatment may be affecting the neuronal response through the differential regulation of cAMP in growth cones.

### Axonal Netrin-1 induces local and long-range increase in calcium transients

**Figure 6:**
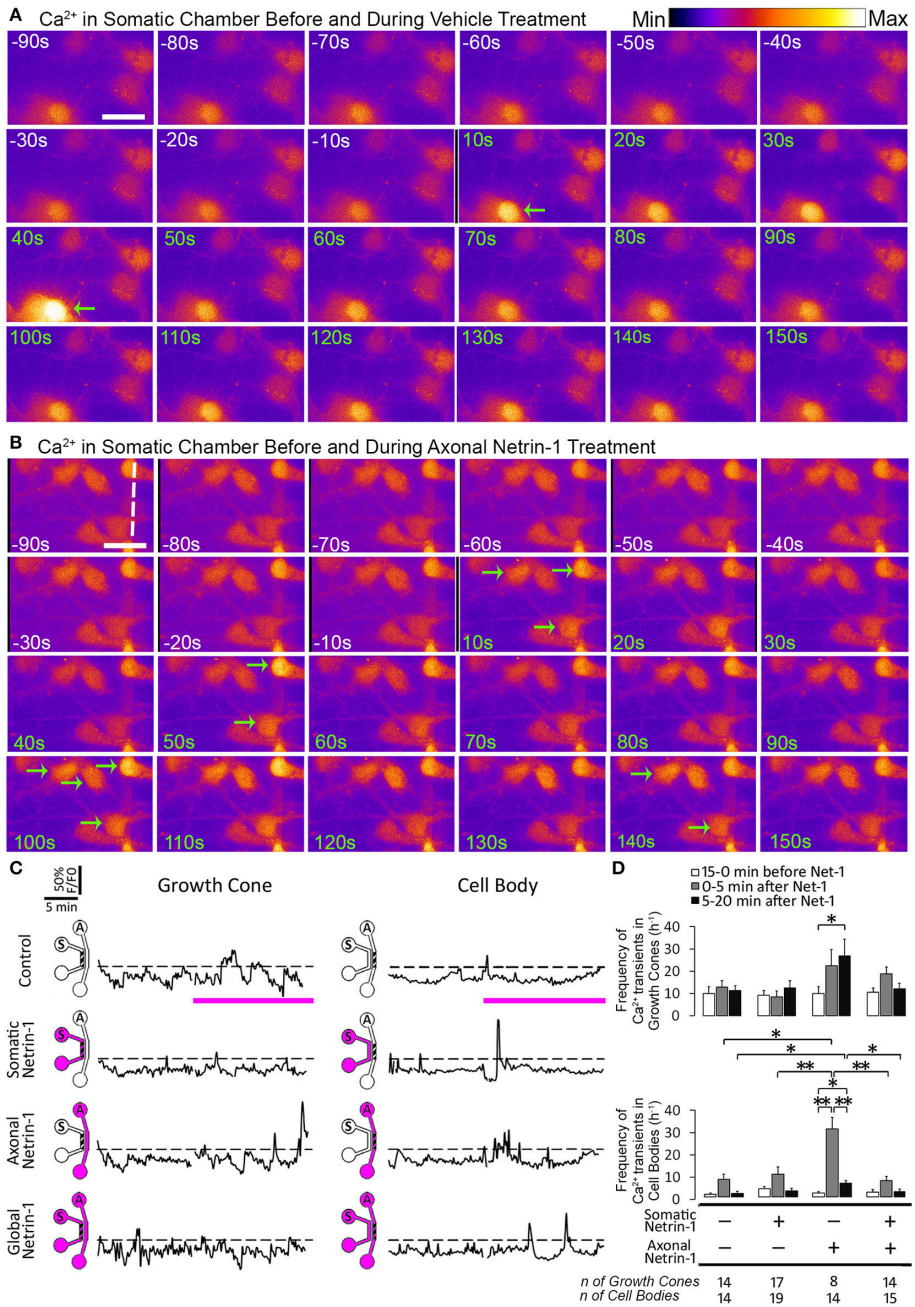
**Axonal but not global Netrin-1 induces local and long-range changes in calcium activity. (A,B)** Calcium activity in cell bodies before (white time overlay) and after (green time overlay) adding vehicle **(A)** or 1.0 μg ml^−1^ Netrin-1 **(B)** into axonal compartment at *t* = 0. Green arrows point at cell bodies that start firing. Broken line indicates the beginning of microchannels. Scale bar = 20 μm. **(C)** Representative calcium activity traces in response to treatment (pink bars) with vehicle (control) or with 1.0 μg ml^−1^ Netrin-1. Signals (*F*) exceeding 20% of the baseline fluorescence (*F0*; dashed lines) were considered positive. The schematics show which compartment, somatic (*S*) or axonal (*A*), underwent Netrin-1 treatment (pink) and which compartment was imaged (hatch). **(D)** Frequency of calcium transients (mean ± s.e.m.); *n* is the number of individual measurements from *N* ≥ 2 distinct cultures (Table S6); ^*^p < 0.05, ^**^p < 0.01.

Since Ca^2+^ is a known regulator of cyclic nucleotide activity, we tested if local and global Netrin-1 treatments affected Ca^2+^ dynamics. We performed time-lapse imaging of calcium dye-loaded growth cones and cell bodies to determine frequency of local Ca^2+^ transients (*f*_Ca_) before and after Netrin-1 (or vehicle) application (Figures 6A,B; Movie S2). Somatic and global Netrin-1 failed to change *f*_Ca_ in growth cones. In contrast, axonal Netrin-1 increased *f*_Ca_ in growth cones from 10 h^−1^ to 22 h^−1^ in the first 5 min and to 27 h^−1^ in the next 15 min (Figures 6C,D). Similarly, when we quantified *f*_Ca_ in cell bodies, somatic and global Netrin-1 failed to change *f*_Ca_, whereas axonal Netrin-1 caused *f*_Ca_ to increase from 1 h^−1^ to 30 h^−1^ in the first 5 min, which dropped to 6 h^−1^ in the next 15 min. These results strongly suggest that Netrin-1 locally activates calcium signaling in growth cones and that this signal is retrogradely propagated to cell bodies. In turn, the co-stimulation of growth cones and cell bodies (global treatment) suppressed Netrin-1-induced *f*_Ca_ increase in all regions.

### Ryanodine receptor activity is required for Netrin-1-mediated DCC membrane insertion

**Figure 7:**
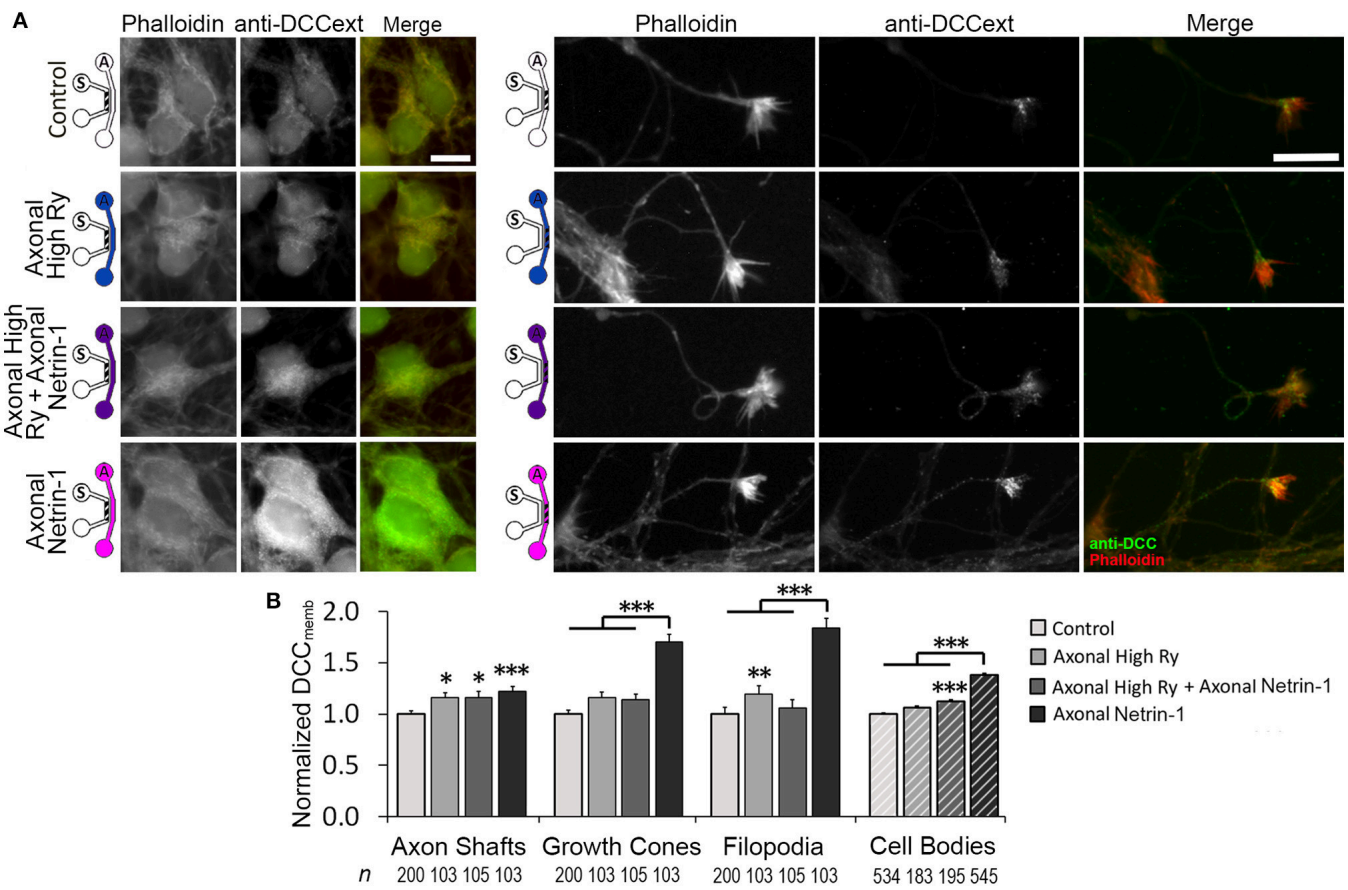
**Calcium efflux from internal stores is necessary for Netrin-1-driven DCC membrane insertion. (A)** Membranous DCC (DCC_memb_) staining intensity after 25 min of axonal treatments, as shown by the schematics: with vehicle (control; PBS for Netrin-1, water for Ryanodine), 100 μM ryanodine (High Ry; blue), 1.0 μg ml^−1^ Netrin-1 (pink), or combined (purple). The hatch shows imaging site—axonal (*A*) or somatic (*S*). Scale bars = 10 μm. **(B)** DCC staining intensity (mean ± s.e.m.) was measured in the ROIs in the axonal (solid fill) and somatic (hatch fill) compartments, and normalized with the control signal. *n* is the number of individual measurements from *N* ≥ 3 independent experiments (Table S7); statistical significance compared to controls unless indicated otherwise; ^*^p < 0.05, ^**^p < 0.01, ^***^p < 0.001. Axonal compartment data for axonal Netrin-1 treatment are re-presented with permission from Blasiak et al. (2015). Copyright 2015 American Chemical Society.

Axonal Netrin-1 treatment increased somatic DCC_memb_ and *f*_Ca_. These observations are suggestive of retrograde signal propagation from the distal axons. Netrin-1 has been shown to activate ryanodine receptors (RyRs) and increase calcium efflux from the ER, which in turn modulates DCC_memb_. RyR-mediated calcium release can propagate along the ER, spreading the signal to distant subcellular regions. We hypothesized that Netrin-1 evokes calcium efflux through RyRs in the growth cones, which propagates toward the cell bodies and mediates the DCC insertion into their plasma membranes. To test this hypothesis, we exposed distal axons to 1.0 μg/ml Netrin-1 for 25 min and simultaneously inhibited their RyRs with 100 μM ryanodine (high Ry). As the treatments were applied only to the axonal compartment, the cell bodies were not directly exposed to Netrin-1 and had intact RyRs. We analyzed DCC_memb_ in distal axons and in cell bodies (Figure 7): Blocking axonal RyRs alone did not affect DCC_memb_ in growth cones or cell bodies. However, it completely blocked the axonal Netrin-1-induced DCC_memb_ increase in growth cones, and suppressed the axonal Netrin-1-induced DCC_memb_ increase in cell bodies (to 1/3 of its value observed in neurons with intact axonal RyRs). DCC_memb_ in axon shafts and filopodia increased after RyRs inhibition alone. These observations suggest that RyRs-mediated calcium release from the ER plays a role in both, a local and long-range DCC dynamics in response to Netrin-1, and highlight the subcellular heterogeneity in DCC membrane insertion.

## Discussion

Our experimental platform integrating microfluidics with molecular and optical techniques facilitates studying neuron behavior in development and disease. Firstly, the study provides mechanistic details on Netrin-1-induced direct (local) and indirect (long-range) effects on neural signaling and axon guidance. Cortical neurons express Netrin-1 and DCC along their entire lengths (Bouchard et al., 2008); thus, cell bodies are exposed to autocrine Netrin-1, confirming the necessity to understand the effect of Netrin-1 on cell bodies to decipher its role in distal axon growth. Secondly, while during development neurons move through Netrin-1 gradients gradually, under pathological conditions, e.g., brain ischemia (Tsuchiya et al., 2007) or spinal cord injury (Manitt et al., 2006) Netrin-1 levels change much more rapidly. Similarly, as Netrin-1 is considered a therapeutic target (Wu et al., 2008; Masuda et al., 2009), neurons may be exposed to Netrin-1 onset outside the physiological range. Understanding the neuronal response to global and local Netrin-1 treatment as applied in this study not only broadens our knowledge of nervous system development, but may also help design novel therapeutic strategies.

### Neuronal cell bodies respond to Netrin-1

The evidence for Netrin-1 response of cell bodies comes from two sets of observations. First, somatic Netrin-1 increased the total DCC level in the distal axons in the axonal compartment. These axons could not sense Netrin-1 directly—the signal had to propagate from the somatic compartment. The second set of evidence stems from comparing axonal and global Netrin-1 treatments: (i) global Netrin-1 induced a more pronounced decrease in distal axon speed compared to axonal Netrin-1; (ii) global Netrin-1 blocked the increase in DCC_memb_ in growth cones, induced by axonal Netrin-1; (iii) global Netrin-1 suppressed the axonal Netrin-1-induced cAMP response; (iv) lastly, global Netrin-1 blocked the Ca^2+^ increase in growth cones and in cell bodies, both induced by axonal Netrin-1.

### Netrin-1 slows down axon elongation

Our axon velocity measurements are in agreement with previous reports on axonal growth in dissociated cortical neuron cultures (Li et al., 2009) and with axon growth rates *in vivo* (Ramón y Cajal, 1928). Netrin-1 is reported predominantly as an attractant for cortical neurons (Bouchard et al., 2008); however, several studies including ours demonstrated its repulsive effects (Powell et al., 2008; Blasiak et al., 2015). Somatic Netrin-1 treatment alone did not affect distal axon growth—signal that had propagated from the cell bodies contributed to axon elongation only when Netrin-1 was present in the axon microenvironment. The difference between the effects on speed and velocity suggests that different mechanisms were evoked by axonal and global treatments. Neurons that experience Netrin-1 locally at their growth cones may have normal cytoskeletal dynamics (hence minor decrease in speed), but an altered directional persistence (hence major decrease in velocity). On the other hand, neurons that experience Netrin-1 globally may have altered cytoskeletal dynamics resulting in the reduction of axon speed and consequently of axon velocity. The directional persistence has been proposed as a link between rapid intracellular signaling events and motility events that occur in much longer time scales (Weiger et al., 2010). Further experiments are required to explore directional persistence in the neuronal context, where intracellular distances are much greater compared to motile cells such as fibroblasts. Another potential explanation for the differences observed between axonal and global treatments is related to axon branching. Netrin-1 has been reported to induce *de novo* branch formation in cortical neurons, which leads to increased competition between branches and may affect axonal growth (Dent et al., 2004). We did not observe *de novo* branch formation during the 90 min Netrin-1 treatment; however, if existing branches of an axon are in different microfluidic compartments they may be competing. Considering the low probability of this arrangement, the observed differences cannot be explained solely by such competition.

### Netrin-1 regulates membranous and total DCC levels

DCC was identified as a tumor suppressor gene (Fearon et al., 1990) before the discovery that it encodes a Netrin-1 receptor (Keino-Masu et al., 1996). DCC has been classified as a dependence receptor, i.e., it induces apoptosis in the absence of its ligand (Mehlen et al., 1998). Accordingly, a dual action mechanism has been proposed: (i) axonal guidance *via* chemotropic activity of Netrin-1, and (ii) apoptotic induction in neurons/axons grown away from the region of Netrin-1 availability (Mehlen and Furne, 2005). These theories have recently raised questions, as they are inconsistent with the observations made using Netrin-1 loss-of-function mice (Bin et al., 2015). In our hands, neurons with isolated distal axons did not show any signs of degeneration in response to Netrin-1, which contradicts the Netrin-1 dual mechanism hypothesis. However, considering that cortical neurons secrete Netrin-1 along their entire lengths (Bouchard et al., 2008), distal axons may be exposed to sufficiently high levels of endogenous Netrin-1 that occupies their membranous DCC to prevent apoptotic induction.

Our results suggest that neurons sense Netrin-1 along their entire lengths and adjust their DCC_memb_ accordingly. Two lines of evidence support this argument: (i) global Netrin-1 canceled the increase in DCC_memb_ in growth cones and filopodia of distal axons induced by axonal Netrin-1; (ii) axonal Netrin-1 induced an elevation in the DCC_memb_ in cell bodies that was twice the elevation in response to directly applied (somatic or global) Netrin-1. DCC_memb_ changes were partially independent of DCC_total_. In distal growth cones, all modes of Netrin-1 treatment increased DCC_total_ at 90 min, while only axonal Netrin-1 elevated DCC_memb_ at 25 min, which returned to baseline at 90 min. In cell bodies, on the other hand, global and somatic, but not axonal Netrin-1 decreased DCC_total_, while all modes of Netrin-1 treatment elevated DCC_memb_ at 25 min, and global and somatic, but not axonal Netrin-1 decreased DCC_total_ below the baseline level at 90 min. These observations suggest two interdependent means of DCC regulation by Netrin-1: (i) the size of DCC cytosolic pool and (ii) the dynamics of DCC cycling on and off the plasma membrane. While the changes in DCC_total_ occurred over long time scales, DCC_memb_ increases were transient in nature. It is plausible to think that membranous DCC underwent internalization due to neuronal adaptation to Netrin-1 (Piper et al., 2005).

Further analysis of DCC_memb_ increase in cell bodies in response to axonal Netrin-1 revealed intriguing observations. First, it suggests that DCC_memb_ was predominantly controlled by the local axonal exposure to Netrin-1, similar to what has been reported for the neuronal response to Slit (Guan et al., 2007). Second, signal transduction from the axonal compartment to the somatic was very rapid: a significant DCC_memb_ increase was observed as early as 5 min after Netrin-1 treatment, consistent with the local DCC dynamics in axons (Blasiak et al., 2015). Lastly, DCC_memb_ increase was uniform within the somatic compartment despite the fact that only a minor fraction of cell bodies had axons in the axonal compartment, hinting to a potential intercellular propagation mechanism in the somatic compartment. Additional studies are required to confirm and explore these phenomena.

DCC is known to modulate the extent of Netrin-1-induced axon turning and elongation responses (Bouchard et al., 2008). In our hands, DCC_memb_ was not correlated with axon velocity: both axonal and global Netrin-1 decreased axon velocity, whereas only axonal Netrin-1 induced DCC_memb_ increase in growth cones. Additionally, axon slowdown continued for at least 90 min—long after DCC_memb_ in growth cones returned to baseline. Cortical neurons have been shown to express UNC5, another Netrin-1 receptor (Blasiak et al., 2015). It is therefore likely that the axon growth is regulated by an interplay between different Netrin-1 receptors present on the membrane (Tojima et al., 2011), which calls for further studies.

### Interplay between second messengers in the growth cone

The influx of extracellular Ca^2+^ is required for Netrin-1-induced repulsive and attractive growth cone turning (Ming et al., 1997) and axon elongation rate changes (Blasiak et al., 2015). Downstream cAMP elevation leads to further Ca^2+^ influx (Nishiyama et al., 2003) and promotes RyRs-mediated, calcium induced calcium release (CICR) (Ooashi et al., 2005). Consequently, high [Ca^2+^]_i_ stimulates cAMP production (Willoughby and Cooper, 2007). Accordingly, our results show that the local Netrin-1 stimulation of growth cones caused a temporary increase in cAMP and a substantial increase in *f*_Ca_, where the latter was sustained even after cAMP returned to the baseline level. The co-stimulation of cell bodies (global Netrin-1) suppressed the increases in both cAMP and *f*_Ca_. Interestingly, CICR and cAMP have been shown to be reduced by increased cGMP activity (Tojima et al., 2009); however we did not detect a statistically significant cGMP increase after global Netrin-1 treatment. Taken together, our results show that axonal and global Netrin-1 treatments differently modulate cyclic nucleotides and calcium signaling. The interdependence in the second messenger system needs to be further tested.

### Signals downstream of Netrin-1 propagate in retrograde and anterograde directions

Our results suggest that Ca^2+^ activity plays an important role in the retrograde signal propagation downstream of Netrin-1. This observation is not unprecedented: exposing growth cones of *Xenopus* spinal neurons to Netrin-1 caused a [Ca^2+^]_i_ rise in cell bodies, whose magnitude was *ca*. half of the [Ca^2+^]_i_ rise measured in growth cones (Hong et al., 2000). Interestingly, when the same research group performed voltage-clamp recordings, they observed that Netrin-1-evoked currents in growth cones were spatially restricted and did not propagate to cell bodies (Nishiyama et al., 2003). Therefore, mechanisms other than membrane potential shifts had to be involved in the Netrin-1-mediated retrograde Ca^2+^ signal propagation. Our results show that the Ca^2+^ signal propagation after axonal Netrin-1 stimulation was accompanied by an increase in DCC_memb_ in cell bodies. This is consistent with previous reports showing that the elevation of [Ca^2+^]_i_ through the activation of RyRs brings DCC to the membrane (Blasiak et al., 2015). Our results also show that the calcium activity in cell bodies is not affected by somatic or global Netrin-1 treatments. As CICR is a key component in the calcium wave propagation system (Verkhratsky, 2002), the elevation of cAMP level in response to axonal Netrin-1 may have activated CICR, which, in turn, may have activated the retrograde signal propagation from growth cones to cell bodies. On the other hand, the inhibition of CICR in growth cones (as suggested by low *f*_Ca_) by global Netrin-1 may have suppressed this retrograde signal propagation. Accordingly, DCC_memb_ in growth cones after global treatment—similar to somatic treatment—was lower than DCC_memb_ after axonal treatment. Supporting the putative role of CICR in the retrograde signal propagation, when we inhibited CICR in growth cones by locally blocking RyRs, axonal Netrin-1 induced a significantly lower increase in the DCC_memb_ in cell bodies. This is in agreement with the observation that CICR mediates [Ca^2+^]_i_ increase in cell bodies after stimulation of growth cones with Slit (Guan et al., 2007).

The differential effects of global vs. axonal Netrin-1 treatments suggested a very rapid (seconds) signal propagation from cell bodies to growth cones that affected cyclic nucleotide activity. The mechanism of this anterograde communication is unclear; however, its timeframe is reminiscent of an action potential. This notion is in agreement with the studies that describe how Netrin-1 induces depolarization (chemo-attraction) or hyperpolarization (chemo-repulsion) of *Xenopus* spinal neuron membranes, where the latter increases cGMP (Nishiyama et al., 2008). Furthermore, action potentials have been shown to mediate long-range signaling downstream of nerve growth factor (which also regulates axonal growth)—its local application evokes action potentials that inhibit axon outgrowth in distant subcellular regions (Singh and Miller, 2005). Similar mechanisms may control long-range cyclic nucleotide activity (Hutchins, 2010). Total DCC measurements also suggested anterograde signal propagation: somatic Netrin-1 increased DCC_total_ in distal growth cones, but decreased it in cell bodies. It is unclear whether DCC was locally expressed in axons (and locally degraded in cell bodies) during the 90 min treatment period, or was actively transported from cell bodies to growth cones. Potential mechanisms, including Ca^2+^ waves, membrane potential shifts, and active axonal transport, need to be explicitly tested using fluidically isolated axons.

## Conclusions

**Figure 8:**
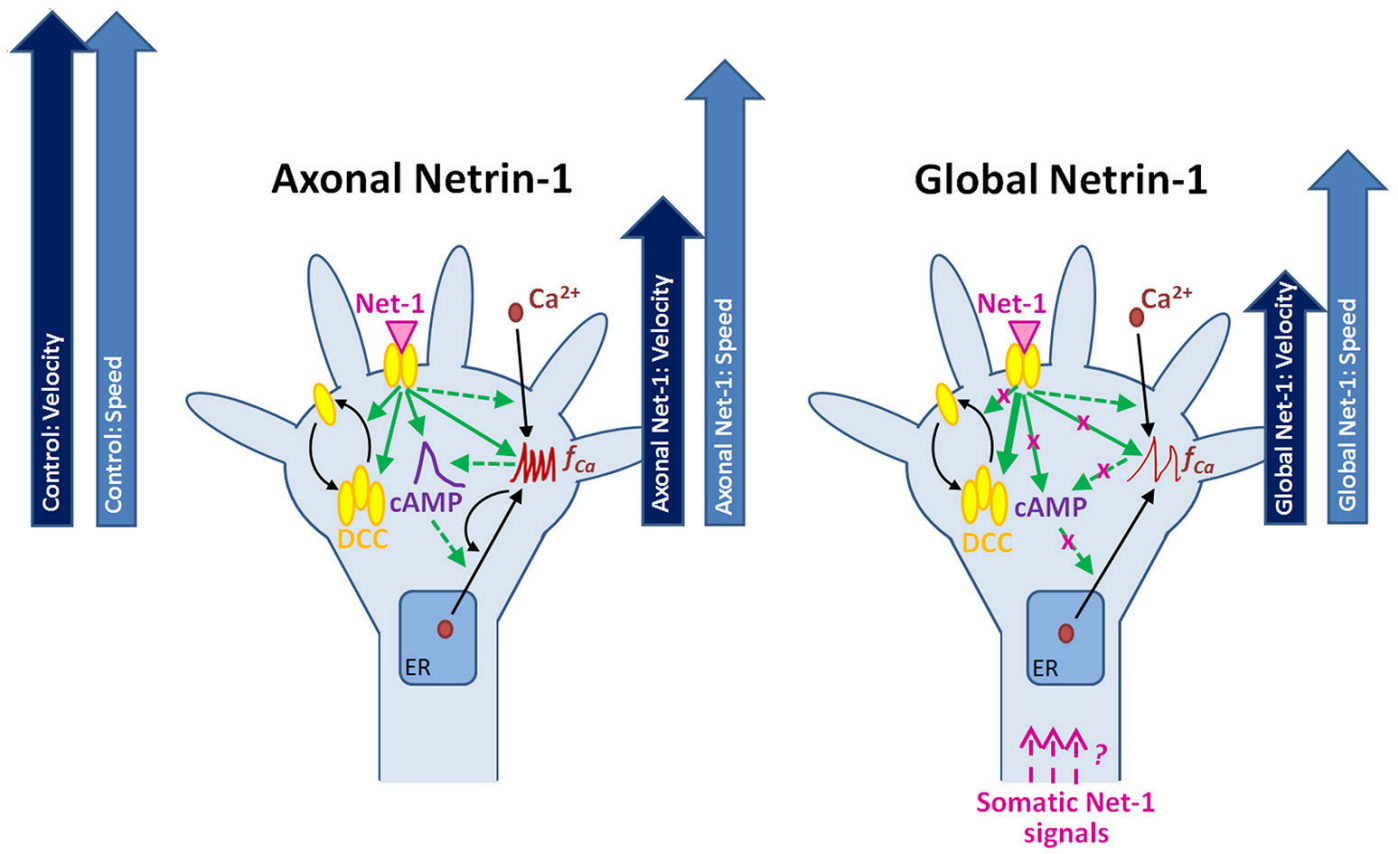
**Putative mechanisms for the neuronal response to locally and globally applied Netrin-1**. Axonal and global Netrin-1 treatments differentially regulate membranous and total DCC levels, and the dynamics of second messengers. Arrows indicate the character and strength of the change as measured in our experiments (solid lines) and based on the literature (broken lines). Axonal Netrin-1 increases cAMP level, the frequency of Ca^2+^ transients and the total and membranous DCC levels in growth cones. cAMP supports Ca^2+^ efflux from the endoplasmic reticulum (ER); high level of Ca^2+^ leads to calcium-induced calcium release (CICR). Global Netrin-1 increases total DCC levels, but not membranous DCC levels, cAMP or the frequency of calcium transients. CICR is inhibited. Axonal Netrin-1 slightly affects the axon speed, and severely decreases axon velocity (block arrows). Global Netrin-1 significantly decreases both, axon speed and velocity. Differences in axonal and global Netrin-1 responses suggest that neurons sense Netrin-1 along their entirety and alter their response accordingly; however, the mechanisms of anterograde propagation of Netrin-1-induced signals are unknown.

Neuronal response to Netrin-1 is spatially controlled, i.e., downstream signaling molecules form concentration gradients across the growth cone and along the axon. The bifunctional character of Netrin-1 and the complexity of its spatio-temporal signaling call for research tools that provide precise control over subcellular microenvironments. Using microfluidic compartmentalization, we demonstrated that, apart from its local effects on the growth cone, Netrin-1 is sensed by the cell bodies leading to changes in the second messengers and alteration of the axon growth response to Netrin-1 (Figure 8). Through direct measurement of cAMP, cGMP and calcium activity in live neurons, our study sheds light on the complex relationship between second messengers and their dynamic responses to Netrin-1 stimulation. Furthermore, we demonstrated that neurons sense Netrin-1 along their entire lengths to adjust their growth. In the nervous system, cell bodies are often located hundreds of microns away from the growth cones; therefore, their fluidic isolation *in vitro* is relevant to the conditions *in vivo*. Our microscopy-based study provides a multitude of novel observations regarding the neuronal response to Netrin-1. The proposed mechanisms linking the second messenger system with the regulation of Netrin-1 receptors and axon elongation behavior requires further experimentation.

## Author contributions

Conceived and designed the experiments: AB, DK, and GUL. Performed the experiments: AB. Analyzed the data: AB and DK. Wrote the paper: AB, DK, and GUL.

## Funding

This work is supported by the Science Foundation Ireland (08/RP1/B1376 and 08/IN1/B2072), by the Nanoremedies Programme funded under the Programme for Research in Third-Level Institutions and co-funded under the European Regional Development Fund, by a Marie Curie Intra-European Fellowship (DK), and by an AXA Research Fund Doctoral Fellowship (AB).

### Conflict of interest statement

The authors declare that the research was conducted in the absence of any commercial or financial relationships that could be construed as a potential conflict of interest.

## Abbreviations

[Ca^2+^]_e_: extracellular Ca^2+^ concentration
[Ca^2+^]_i_: intracellular Ca^2+^ concentration
CICR: Calcium-Induced Calcium Release
DCC: Deleted in Colorectal Cancer
*f*_Ca_: frequency of Ca^2+^ transients
Ry: ryanodine
RyRs: ryanodine receptors
FRET: Förster Resonance Energy Transfer.

## References

[B1] Albus C. A. Rishal I. Fainzilber M. (2013). Cell length sensing for neuronal growth control. Trends Cell Biol. 23, 305–310. 10.1016/j.tcb.2013.02.001 23511112

[B2] Averaimo S. Nicol X. (2014). Intermingled cAMP, cGMP and calcium spatiotemporal dynamics in developing neuronal circuits. Front. Cell. Neurosci. 8:376. 10.3389/fncel.2014.00376 25431549 PMC4230202

[B3] Bartoe J. L. McKenna W. L. Quan T. K. Stafford B. K. Moore J. A. Xia J.. (2006). Protein interacting with C-kinase 1/protein kinase Cα-mediated endocytosis converts netrin-1-mediated repulsion to attraction. J. Neurosci. 26, 3192–3205. 10.1523/JNEUROSCI.3469-05.2006 16554470 PMC6674106

[B4] Bin J. M. Han D. Lai Wing Sun K. Croteau L. P. Dumontier E. Cloutier J. F.. (2015). Complete loss of Netrin-1 results in embryonic lethality and severe axon guidance defects without increased neural cell death. Cell Rep. 12, 1099–1106. 10.1016/j.celrep.2015.07.028 26257176

[B5] Blasiak A. Lee G. U. Kilinc D. (2015). Neuron subpopulations with different elongation rates and DCC dynamics exhibit distinct responses to isolated Netrin-1 treatment. ACS Chem. Neurosci. 6, 1578–1590. 10.1021/acschemneuro.5b00142 26190161

[B6] Börner S. Schwede F. Schlipp A. Berisha F. Calebiro D. Lohse M. J.. (2011). FRET measurements of intracellular cAMP concentrations and cAMP analog permeability in intact cells. Nat. Protoc. 6, 427–438. 10.1038/nprot.2010.198 21412271

[B7] Bouchard J. F. Horn K. E. Stroh T. Kennedy T. E. (2008). Depolarization recruits DCC to the plasma membrane of embryonic cortical neurons and enhances axon extension in response to netrin-1. J. Neurochem. 107, 398–417. 10.1111/j.1471-4159.2008.05609.x 18691385

[B8] Bouchard J. F. Moore S. W. Tritsch N. X. Roux P. P. Shekarabi M. Barker P. A.. (2004). Protein kinase A activation promotes plasma membrane insertion of DCC from an intracellular pool: a novel mechanism regulating commissural axon extension. J. Neurosci. 24, 3040–3050. 10.1523/JNEUROSCI.4934-03.2004 15044543 PMC6729852

[B9] Bradford D. Cole S. J. Cooper H. M. (2009). Netrin-1: diversity in development. Int. J. Biochem. Cell Biol. 41, 487–493. 10.1016/j.biocel.2008.03.014 18455953

[B10] Dent E. W. Barnes A. M. Tang F. Kalil K. (2004). Netrin-1 and semaphorin 3A promote or inhibit cortical axon branching, respectively, by reorganization of the cytoskeleton. J. Neurosci. 24, 3002–3012. 10.1523/JNEUROSCI.4963-03.2004 15044539 PMC6729836

[B11] Fazeli A. Dickinson S. L. Hermiston M. L. Tighe R. V. Steen R. G. Small C. G.. (1997). Phenotype of mice lacking functional Deleted in colorectal cancer (Dcc) gene. Nature 386, 796–804. 10.1038/386796a0 9126737

[B12] Fearon E. R. Cho K. R. Nigro J. M. Kern S. E. Simons J. W. Ruppert J. M.. (1990). Identification of a chromosome 18q gene that is altered in colorectal cancers. Science 247, 49–56. 10.1126/science.2294591 2294591

[B13] Furne C. Rama N. Corset V. Chedotal A. Mehlen P. (2008). Netrin-1 is a survival factor during commissural neuron navigation. Proc. Natl. Acad. Sci. U.S.A. 105, 14465–14470. 10.1073/pnas.0803645105 18796601 PMC2567197

[B14] Glynn M. W. McAllister A. K. (2006). Immunocytochemistry and quantification of protein colocalization in cultured neurons. Nat. Protoc. 1, 1287–1296. 10.1038/nprot.2006.220 17406413

[B15] Gorbunova Y. V. Spitzer N. C. (2002). Dynamic interactions of cyclic AMP transients and spontaneous Ca^2+^ spikes. Nature 418, 93–96. 10.1038/nature00835 12097913

[B16] Guan C. B. Xu H. T. Jin M. Yuan X. B. Poo M. M. (2007). Long-range Ca^2+^ signaling from growth cone to soma mediates reversal of neuronal migration induced by slit-2. Cell 129, 385–395. 10.1016/j.cell.2007.01.051 17448996

[B17] Hong K. Nishiyama M. Henley J. Tessier-Lavigne M. Poo M. (2000). Calcium signalling in the guidance of nerve growth by netrin-1. Nature 403, 93–98. 10.1038/47507 10638760

[B18] Hutchins B. I. (2010). Competitive outgrowth of neural processes arising from long-distance cAMP signaling. Sci. Signal. 3:jc1. 10.1126/scisignal.3118jc1 20407121 PMC3329774

[B19] Keino-Masu K. Masu M. Hinck L. Leonardo E. D. Chan S. S. Culotti J. G.. (1996). Deleted in Colorectal Cancer (DCC) encodes a netrin receptor. Cell 87, 175–185. 10.1016/S0092-8674(00)81336-7 8861902

[B20] Kilinc D. Blasiak A. O'Mahony J. J. Lee G. U. (2014). Low piconewton towing of CNS axons against diffusing and surface-bound repellents requires the inhibition of motor protein-associated pathways. Sci. Rep. 4:7128. 10.1038/srep07128 25417891 PMC4241520

[B21] Kilinc D. Peyrin J. M. Soubeyre V. Magnifico S. Saias L. Viovy J. L.. (2011). Wallerian-like degeneration of central neurons after synchronized and geometrically registered mass axotomy in a three-compartmental microfluidic chip. Neurotox. Res. 19, 149–161. 10.1007/s12640-010-9152-8 20162389 PMC3006648

[B22] Kim T. H. Lee H. K. Seo I. A. Bae H. R. Suh D. J. Wu J.. (2005). Netrin induces down-regulation of its receptor, Deleted in Colorectal Cancer, through the ubiquitin-proteasome pathway in the embryonic cortical neuron. J. Neurochem. 95, 1–8. 10.1111/j.1471-4159.2005.03314.x 16181408 PMC2683579

[B23] Lai Wing Sun K. Correia J. P. Kennedy T. E. (2011). Netrins: versatile extracellular cues with diverse functions. Development 138, 2153–2169. 10.1242/dev.044529 21558366

[B24] Li L. Hutchins B. I. Kalil K. (2009). Wnt5a induces simultaneous cortical axon outgrowth and repulsive axon guidance through distinct signaling mechanisms. J. Neurosci. 29, 5873–5883. 10.1523/JNEUROSCI.0183-09.2009 19420254 PMC2697037

[B25] Manitt C. Wang D. Kennedy T. E. Howland D. R. (2006). Positioned to inhibit: netrin-1 and netrin receptor expression after spinal cord injury. J. Neurosci. Res. 84, 1808–1820. 10.1002/jnr.21070 16998900

[B26] Masuda T. Yaginuma H. Sakuma C. Ono K. (2009). Netrin-1 signaling for sensory axons: involvement in sensory axonal development and regeneration. Cell Adh. Migr. 3, 171–173. 10.4161/cam.3.2.7837 19262170 PMC2679878

[B27] Mehlen P. Furne C. (2005). Netrin-1: when a neuronal guidance cue turns out to be a regulator of tumorigenesis. Cell. Mol. Life Sci. 62, 2599–2616. 10.1007/s00018-005-5191-3 16158190 PMC11139161

[B28] Mehlen P. Rabizadeh S. Snipas S. J. Assa-Munt N. Salvesen G. S. Bredesen D. E. (1998). The DCC gene product induces apoptosis by a mechanism requiring receptor proteolysis. Nature 395, 801–804. 10.1038/27441 9796814

[B29] Meijering E. Dzyubachyk O. Smal I. (2012). Methods for cell and particle tracking. Meth. Enzymol. 504, 183–200. 10.1016/b978-0-12-391857-4.00009-4 22264535

[B30] Ming G. L. Song H. J. Berninger B. Holt C. E. Tessier-Lavigne M. Poo M. M. (1997). cAMP-dependent growth cone guidance by netrin-1. Neuron 19, 1225–1235. 10.1016/S0896-6273(00)80414-6 9427246

[B31] Mortimer D. Pujic Z. Vaughan T. Thompson A. W. Feldner J. Vetter I.. (2010). Axon guidance by growth-rate modulation. Proc. Natl. Acad. Sci. U.S.A. 107, 5202–5207. 10.1073/pnas.0909254107 20194766 PMC2841880

[B32] Nairn A. C. Hemmings H. C. Jr. Greengard P. (1985). Protein kinases in the brain. Annu. Rev. Biochem. 54, 931–976. 10.1146/annurev.bi.54.070185.004435 2411213

[B33] Nicol X. Hong K. P. Spitzer N. C. (2011). Spatial and temporal second messenger codes for growth cone turning. Proc. Natl. Acad. Sci. U.S.A. 108, 13776–13781. 10.1073/pnas.1100247108 21795610 PMC3158175

[B34] Nikolaev V. O. Bünemann M. Hein L. Hannawacker A. Lohse M. J. (2004). Novel single chain cAMP sensors for receptor-induced signal propagation. J. Biol. Chem. 279, 37215–37218. 10.1074/jbc.C400302200 15231839

[B35] Nishiyama M. Hoshino A. Tsai L. Henley J. R. Goshima Y. Tessier-Lavigne M.. (2003). Cyclic AMP/GMP-dependent modulation of Ca^2+^ channels sets the polarity of nerve growth-cone turning. Nature 423, 990–995. 10.1038/nature01751 12827203

[B36] Nishiyama M. von Schimmelmann M. J. Togashi K. Findley W. M. Hong K. (2008). Membrane potential shifts caused by diffusible guidance signals direct growth-cone turning. Nat. Neurosci. 11, 762–771. 10.1038/nn.2130 18536712

[B37] Ooashi N. Futatsugi A. Yoshihara F. Mikoshiba K. Kamiguchi H. (2005). Cell adhesion molecules regulate Ca^2+^-mediated steering of growth cones via cyclic AMP and ryanodine receptor type 3. J. Cell Biol. 170, 1159–1167. 10.1083/jcb.200503157 16172206 PMC2171540

[B38] Piper M. Salih S. Weinl C. Holt C. E. Harris W. A. (2005). Endocytosis-dependent desensitization and protein synthesis-dependent resensitization in retinal growth cone adaptation. Nat. Neurosci. 8, 179–186. 10.1038/nn1380 15643427 PMC3682638

[B39] Powell A. W. Sassa T. Wu Y. Tessier-Lavigne M. Polleux F. (2008). Topography of thalamic projections requires attractive and repulsive functions of Netrin-1 in the ventral telencephalon. PLoS Biol. 6:e116. 10.1371/journal.pbio.0060116 18479186 PMC2584572

[B40] Ramón y Cajal S. (1928). Degeneration and Regeneration of the Nervous System. London: Oxford University Press.

[B41] Russwurm M. Mullershausen F. Friebe A. Jäger R. Russwurm C. Koesling D. (2007). Design of fluorescence resonance energy transfer (FRET)-based cGMP indicators: a systematic approach. Biochem. J. 407, 69–77. 10.1042/BJ20070348 17516914 PMC2267402

[B42] Serafini T. Colamarino S. A. Leonardo E. D. Wang H. Beddington R. Skarnes W. C.. (1996). Netrin-1 is required for commissural axon guidance in the developing vertebrate nervous system. Cell 87, 1001–1014. 10.1016/S0092-8674(00)81795-X 8978605

[B43] Shimazaki H. Shinomoto S. (2007). A method for selecting the bin size of a time histogram. Neural Comput. 19, 1503–1527. 10.1162/neco.2007.19.6.1503 17444758

[B44] Singh K. K. Miller F. D. (2005). Activity regulates positive and negative neurotrophin-derived signals to determine axon competition. Neuron 45, 837–845. 10.1016/j.neuron.2005.01.049 15797546

[B45] Taylor A. M. Menon S. Gupton S. L. (2015). Passive microfluidic chamber for long-term imaging of axon guidance in response to soluble gradients. Lab Chip. 15, 2781–2789. 10.1039/c5lc00503e 26000554 PMC4485391

[B46] Tojima T. Hines J. H. Henley J. R. Kamiguchi H. (2011). Second messengers and membrane trafficking direct and organize growth cone steering. Nat. Rev. Neurosci. 12, 191–203. 10.1038/nrn2996 21386859 PMC3133775

[B47] Tojima T. Itofusa R. Kamiguchi H. (2009). The nitric oxide-cGMP pathway controls the directional polarity of growth cone guidance via modulating cytosolic Ca^2+^ signals. J. Neurosci. 29, 7886–7897. 10.1523/JNEUROSCI.0087-09.2009 19535600 PMC6665622

[B48] Tsuchiya A. Hayashi T. Deguchi K. Sehara Y. Yamashita T. Zhang H.. (2007). Expression of netrin-1 and its receptors DCC and neogenin in rat brain after ischemia. Brain Res. 1159, 1–7. 10.1016/j.brainres.2006.12.096 17574219

[B49] Verkhratsky A. (2002). The endoplasmic reticulum and neuronal calcium signalling. Cell Calcium 32, 393–404. 10.1016/S0143416002001896 12543098

[B50] Weiger M. C. Ahmed S. Welf E. S. Haugh J. M. (2010). Directional persistence of cell migration coincides with stability of asymmetric intracellular signaling. Biophys. J. 98, 67–75. 10.1016/j.bpj.2009.09.051 20085720 PMC2800965

[B51] Willoughby D. Cooper D. M. (2007). Organization and Ca^2+^ regulation of adenylyl cyclases in cAMP microdomains. Physiol. Rev. 87, 965–1010. 10.1152/physrev.00049.2006 17615394

[B52] Wu T. W. Li W. W. Li H. (2008). Netrin-1 attenuates ischemic stroke-induced apoptosis. Neuroscience 156, 475–482. 10.1016/j.neuroscience.2008.08.015 18786616

