# Supplementary material for: Neuronal Cell Bodies Remotely Regulate Axonal Growth Response to Localized Netrin-1 Treatment via Second Messenger and DCC Dynamics
Source: Front Cell Neurosci. 2017 Jan 5;10:298. doi: 10.3389/fncel.2016.00298 (PMC5214882; doi:10.3389/fncel.2016.00298)
Supplement: Supplementary file 3 [file DataSheet1.PDF]

Supplementary Material

Neuronal Cell Bodies Remotely Regulate Axonal Growth Response to Localized Netrin-1 Treatment via Second Messenger and DCC Dynamics

Agata Blasiak, Devrim Kilinc*, Gil U Lee*

*** Correspondence:** devrim.kilinc@pasteur-lille.fr; gil.lee@ucd.ie

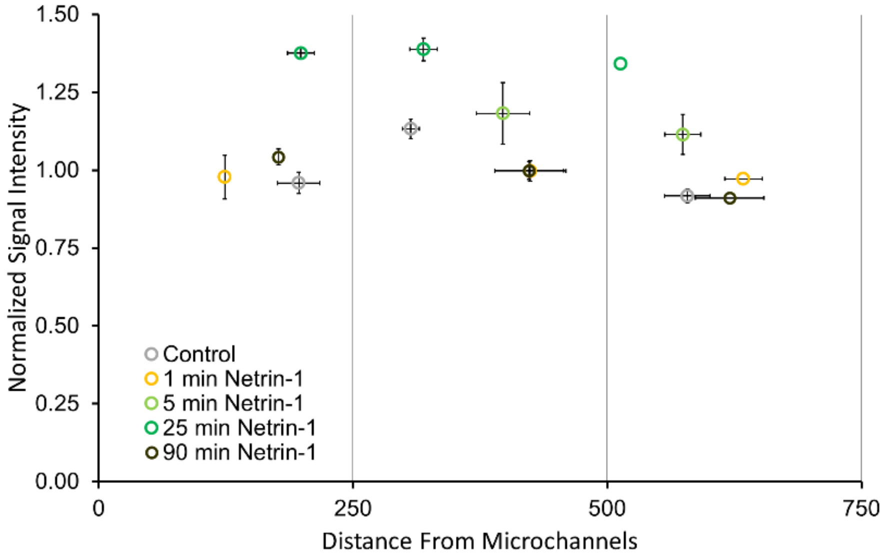

Supplementary Figure 1. DCC$_{memb}$ in cell bodies after axonal Netrin-1 treatment is not correlated with the distance from the microchannels. DCC$_{memb}$ was measured in randomly chosen positions in the somatic compartment after 1–90 min of axonal Netrin-1 or vehicle (control) treatment. Mean intensities (± s.e.m.) were calculated for each third of the width of the somatic compartment. ANOVA; $p > 0.24$ for control; $p > 0.99$ for 1 min and 5 min Netrin-1; $p > 0.90$ for 25 min Netrin-1; $p > 0.06$ for 90 min Netrin-1; n = 14–16 cell bodies.

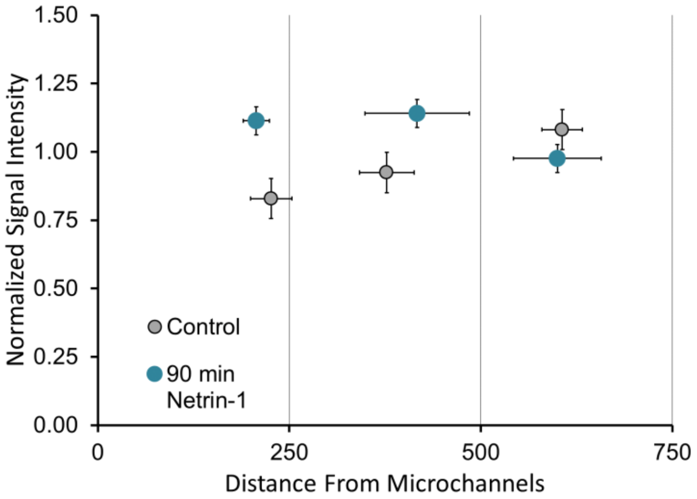

Supplementary Figure 2. DCC$_{total}$ in cell bodies after axonal Netrin-1 treatment is not correlated with the distance from the microchannels. DCC$_{total}$ was measured in randomly chosen positions in the somatic compartment after 90 min of axonal Netrin-1 or vehicle (control) treatment. Mean intensities (± s.e.m.) were calculated for each third of the width of the somatic compartment. ANOVA; $p > 0.13$ for controls; $p > 0.44$ for Netrin-1; n = 9–12 cell bodies.

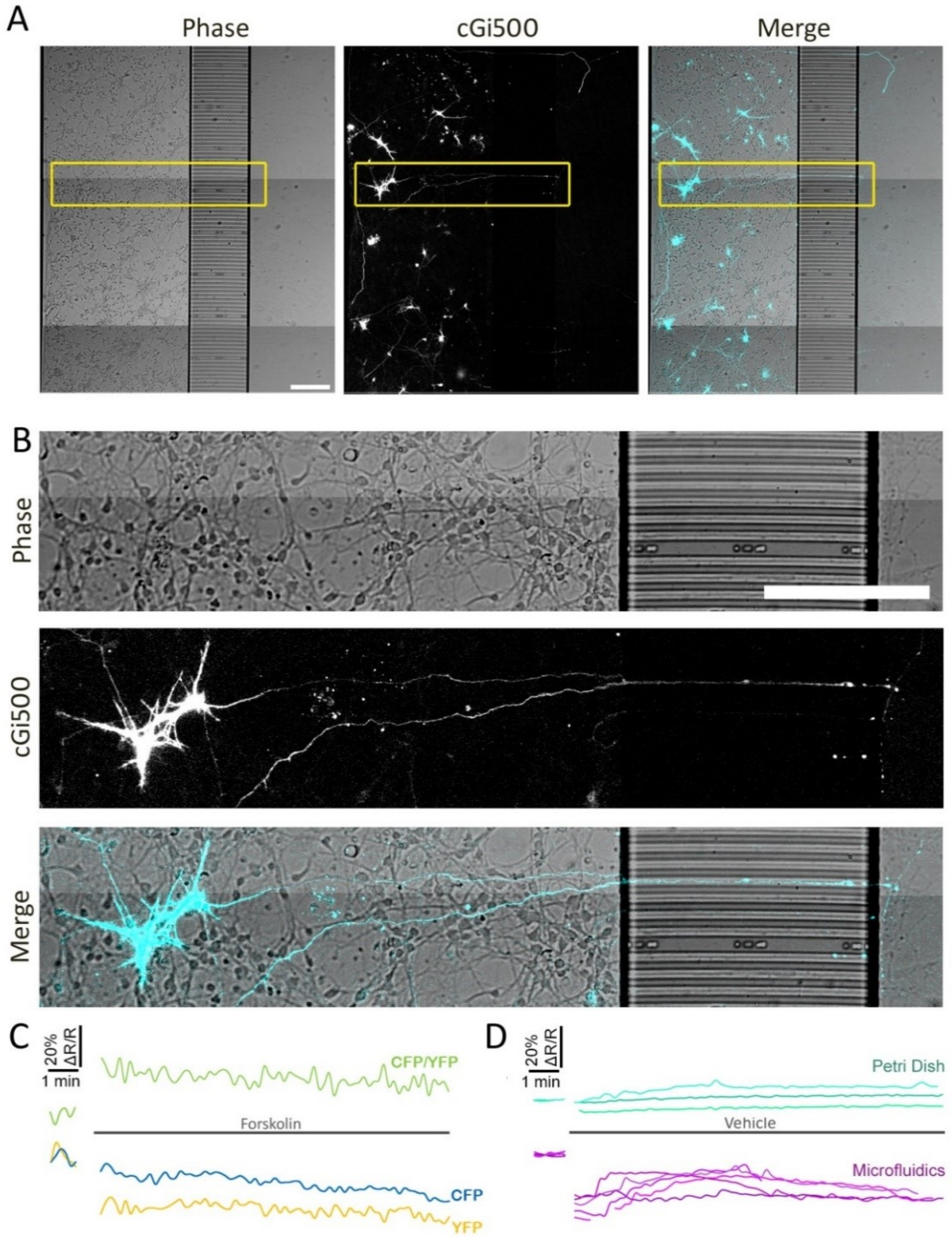

Supplementary Figure 3. Cyclic nucleotide FRET reporters in microfluidics. (**A**) cGi500 transfected neurons growing in the microfluidic. (**B**) The boxed area in (**A**) is magnified to demonstrate transfected cells sending axons through the microchannels. Scale bars = 200 µm. (**C**) Bath application of 10 µM forskolin (adenylyl cyclase agonist) to Epac2 expressing growth cones elevated CFP/YFP ratio, which was correlated with subtle vs. profound decreases in the CFP and YFP signals, respectively. (**D**) Epac2 expressing neurons cultured in microfluidics exhibited decrease of $(\Delta R/R)_{cAMP}$ signal in their cell bodies (individual data traces are shown in different shades of pink) upon inducing flow by applying 10 µl medium with vehicle into one wells of a somatic compartment with 40 µl of medium. This effect was not observed when 40 µl medium with vehicle was applied horizontally to neurons cultured in Petri dish with 160 µl medium (individual data traces are shown in different shades of blue).

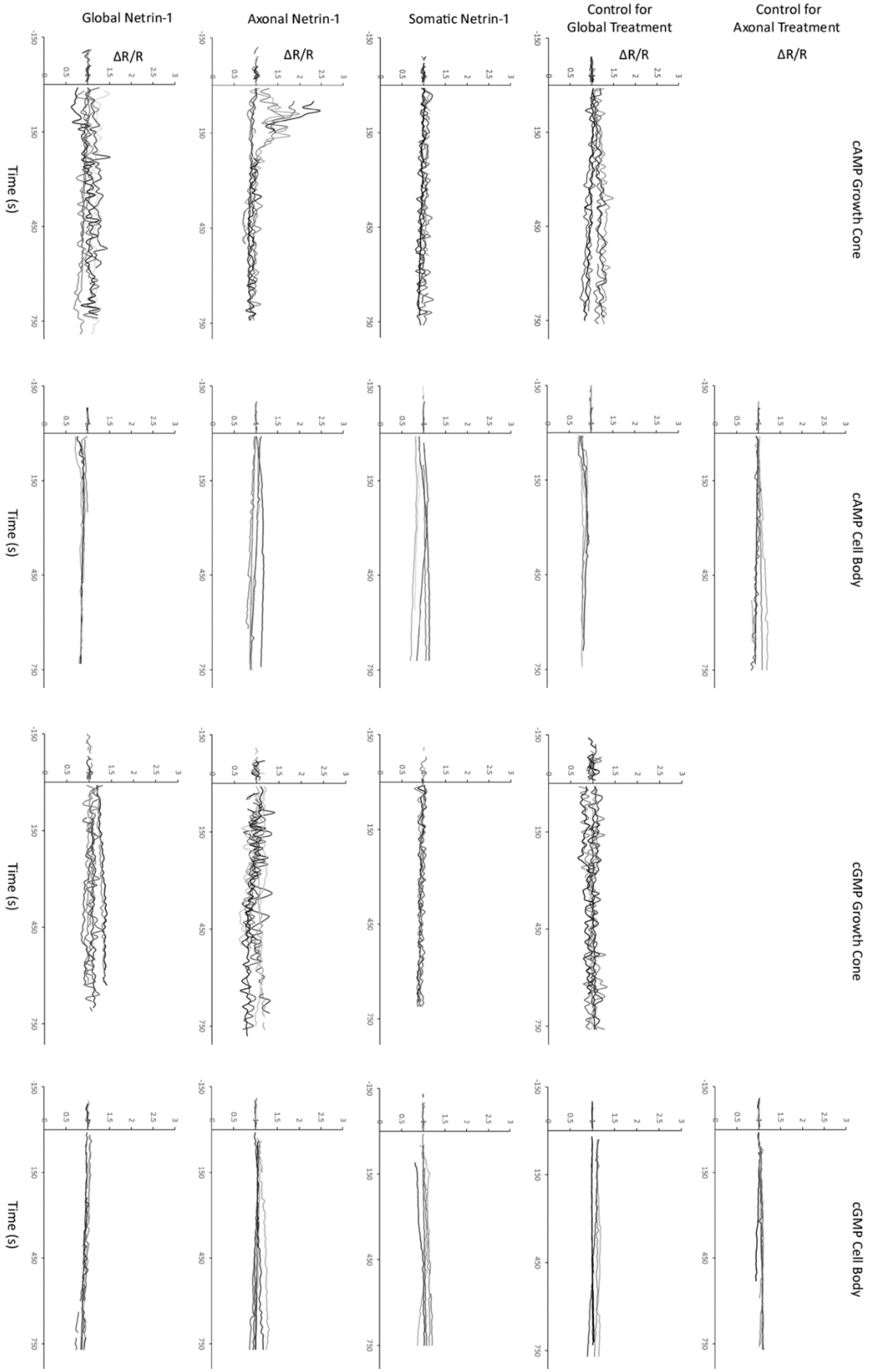
Global Netrin-1
Axonal Netrin-1
Somatic Netrin-1
Control for Global Treatment
Control for Axonal Treatment
ΔR/R
cAMP Growth Cone
cAMP Cell Body
cGMP Growth Cone
cGMP Cell Body
Time (s)

Supplementary Figure 4. Individual data traces for FRET measurements shown in Fig 5. Individual data traces (shown in different shades of grey) of cAMP and cGMP signals in growth cones and in cell bodies in response to treatments (zero time point). CFP:YFP fluorescence intensity ratio (ΔR) was normalized by the mean ratio obtained prior to Netrin-1 or vehicle application (R). Control for axonal treatment means that the vehicle was applied only to the axonal compartment and control for global treatment means that the vehicle was applied globally.

Supplementary Table 1. Numbers of technical and biological (in parentheses) replicates for each experimental condition shown in Figure 1.

	Netrin-1 concentration (μg/L)		
	0.0	0.1	1.0
Control	19 (8)	x	x
Somatic Treatment	x	7 (2)	8 (3)
Axonal Treatment	x	4 (1)	8 (5)
Global Treatment	x	2 (1)	9 (3)

Supplementary Table 2. Numbers of technical and biological (in parentheses) replicates for each experimental condition shown in Figure 2.

	25 min		90 min	
	Cell Bodies	Axons	Cell Bodies	Axons
Control	9(3)	9(3)	6(2)	6(2)
Somatic Treatment	3 (1)	3 (1)	3 (1)	3 (1)
Axonal Treatment	3 (1)	3 (1)	3 (1)	3 (1)
Global Treatment	3 (1)	3 (1)	3 (1)	3 (1)

Supplementary Table 3. Numbers of technical and biological (in parentheses) replicates for each experimental condition shown in Figure 3.

	DCC_{memb} in Cell Bodies				
	Control	1 min	5 min	25 min	90 min
Axonal Treatment	3 (1)	3 (1)	3 (1)	3 (1)	3 (1)

Supplementary Table 4. Numbers of technical and biological (in parentheses) replicates for each experimental condition shown in Figure 4.

	Cell Bodies	Axons
**Control**	4(1)	4(1)
**Somatic Treatment**	3 (1)	3 (1)
**Axonal Treatment**	3 (1)	3 (1)
**Global Treatment**	3 (1)	3 (1)

Supplementary Table 5. Numbers technical and biological (in parentheses) replicates for each experimental condition shown in Figure 5.

	cAMP		cGMP	
	Cell Bodies	Growth Cones	Cell Bodies	Growth Cones
**Control (Global)**	5(2)	5(3)	6(3)	7(5)
**Control (Axonal)**	5(4)	x	5(2)	x
**Somatic Treatment**	5(4)	5(3)	5(4)	4(1)
**Axonal Treatment**	5(3)	4(2)	6(2)	6(4)
**Global Treatment**	5(2)	5(3)	6(2)	6(4)

Supplementary Table 6. Numbers of technical and biological (in parentheses) replicates for each experimental condition shown in Figure 6.

	Cell Bodies	Axons
**Control**	4(3)	6(2)
**Somatic Treatment**	3(2)	6(1)
**Axonal Treatment**	2(2)	3(1)
**Global Treatment**	3(2)	6(2)

Supplementary Table 7. Numbers of technical and biological (in parentheses) replicates for each experimental condition shown in Figure 7.

	Cell Bodies	Axons
**Control**	6(2)	6(2)
**Axonal High Ry**	3 (1)	3 (1)
**Axonal High Ry + Axonal Netrin-1**	3 (1)	3 (1)
**Axonal Netrin-1**	3 (1)	3 (1)
